# Modern Methods of Preserving Meat and Meat Products: A Comprehensive Literature Review

**DOI:** 10.3390/foods15213889

**Published:** 2026-10-30

**Authors:** Mariusz Rudy

**Affiliations:** Department of Agricultural Processing and Commodity Science, Institute of Food Technology and Nutrition, Faculty of Technology and Life Sciences, University of Rzeszów, Zelwerowicza 4, 35-601 Rzeszow, Poland; mrudy@ur.edu.pl; Tel.: +48-(0)-17-785-52-60

**Keywords:** meat preservation, active packaging, high pressure, ultrasound, cold plasma, pulsed light beam, rotating magnetic field, biopreservation, smart freshness indicators, artificial intelligence, machine learning, IoT

## Abstract

In the face of growing consumer demands, tightening legal regulations, and the global problem of food waste, the meat industry is undergoing a dynamic transformation. Traditional, synthetic additive-based preservation methods are being replaced by innovative, integrated technologies. This review article analyzes the latest scientific reports (from 2016–2026) indexed in Scopus, PubMed, Web of Science, and ScienceDirect databases regarding modern methods of meat and meat product preservation. The mechanisms of action and the impact of advanced non-thermal technologies, such as high hydrostatic pressure (HPP), high-intensity ultrasound (HIU), atmospheric pressure cold plasma (ACP), pulsed light (PL), and rotating and oscillating magnetic fields (RMF/OMF), are discussed in detail. The role of biopreservation in the context of the “clean label” trend was also presented, including the use of lactic acid bacteria, bacteriocins, bacteriophages, and nanoencapsulated plant extracts. Furthermore, innovations in materials engineering were analyzed—active packaging releasing antimicrobial substances and intelligent freshness indicators based on natural halochrome dyes. The whole is complemented by an analysis of the impact of Industry 4.0 solutions, including artificial intelligence (AI), machine learning (ML), hyperspectral imaging (HSI), and the Internet of Things (IoT), on cold chain optimization and digital quality management.

## 1. Introduction

Meat and meat products are a key element of the human diet, providing high-quality protein, essential amino acids, B vitamins (especially B_12_), and highly absorbable heme iron. However, due to its high water activity (aw > 0.98), optimal pH for microorganism growth (5.5–6.5), and richness in nutrients, this raw material is one of the most susceptible food products to spoilage [1].

Meat quality degradation occurs in many ways and involves three main mechanisms:

1. Microbiological spoilage—uncontrolled growth of psychrotrophic microflora (e.g., *Pseudomonas* spp., *Brochothrix thermosphacta*, and *Lactobacillus* spp.) and dangerous food pathogens (*Listeria monocytogenes*, *Salmonella enterica*, *Escherichia coli* O157:H7, and *Campylobacter jejuni*), leading to sliminess, unpleasant odor (production of hydrogen sulphide, diketogens, catecholamines) and health hazard [2].
2. Lipid and protein oxidation—autoxidation and photoxidation of polyunsaturated fatty acids (PUFA) lead to the formation of secondary oxidation products (e.g., malondialdehyde—MDA), which cause rancidity, loss of nutritional value and reduced protein digestibility through their aggregation and cross- linking [2].
3. Enzymatic autolysis—endogenous proteases (calpains, cathepsins) and lipases lead to the hydrolysis of muscle tissue, which, although in the initial phase it favors maturation, in the long run reduces the technological quality of the raw material.

**Figure 1:**
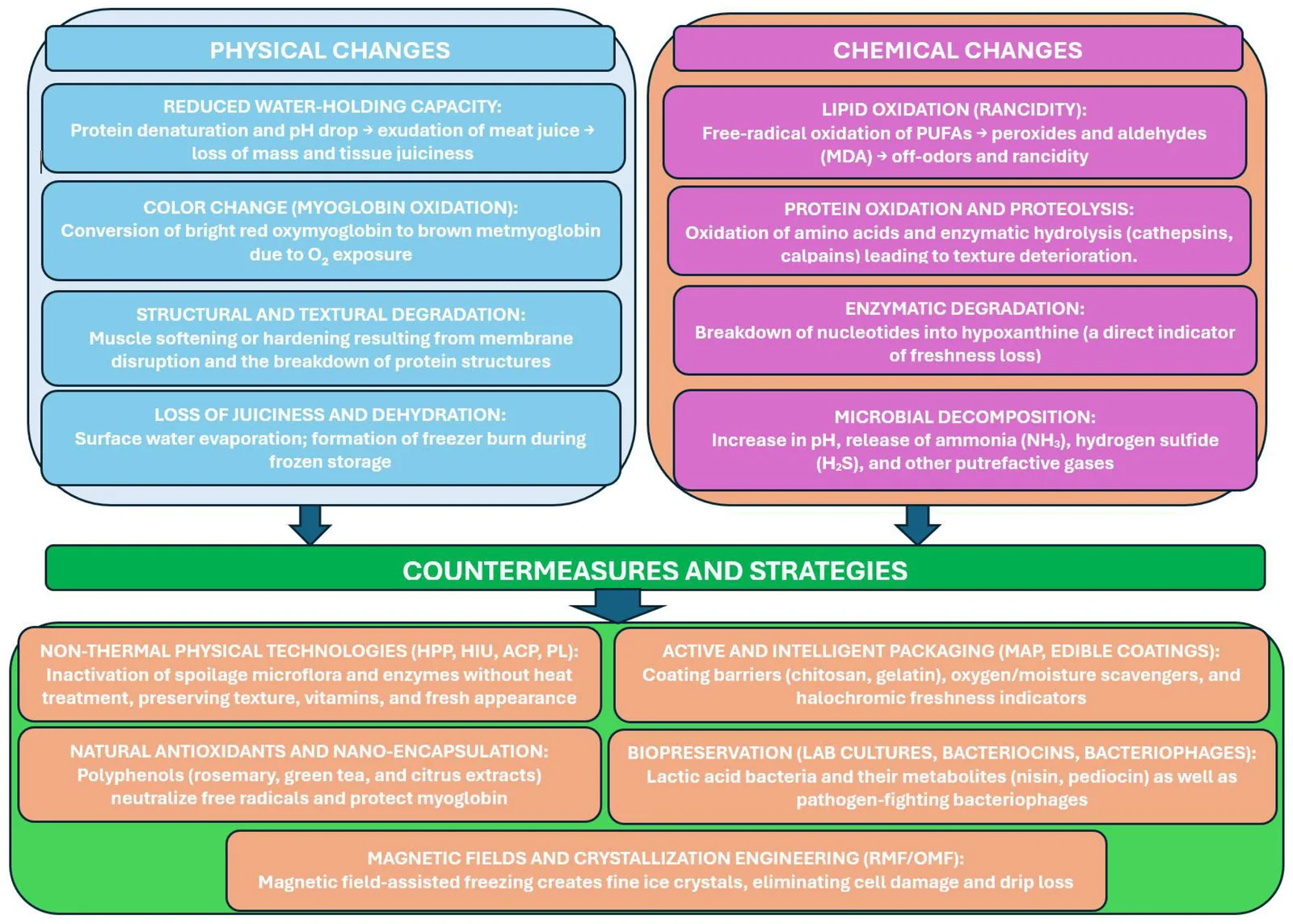
Physicochemical changes occurring in meat during storage and innovative mitigation methods. Prepared by the author based on the following sources: [6,7,8,9,10,11].

According to data from the Food and Agriculture Organization of the United Nations (FAO), meat losses and waste at the processing, distribution and consumption stages amount to up to 20% of global production [3]. Given the high carbon and water footprint associated with livestock farming, extending the shelf life of meat is a priority ecological, economic and technological challenge. For decades, the mainstay of meat preservation has been the use of chemical additives, such as nitrates and nitrites, sulfates, and synthetic antioxidants (BHA—Butylated Hydroxyanisole, BHT—Butylated Hydroxytoluene, TBHQ—Tert-Butylhydroquinone). Although they demonstrate high antimicrobial efficacy (including against *Clostridium botulinum*) and stabilize color, their use is increasingly controversial. Reactions of nitrites with secondary and tertiary amines in the acidic environment of the stomach or during thermal processing lead to the formation of carcinogenic N—nitrosamines [4]. Consumer awareness is currently driving a strong trend toward “clean label”. Consumers expect natural products, free from artificial preservatives, minimally processed, yet characterized by a long shelf life and a high level of microbiological safety. The answer to these mutually exclusive requirements is the hurdle concept created by Leistner, currently under intensive development. It involves the synergistic combination of several mild, sub -lethal preserving factors (e.g., low pH, water activity, non-thermal physical methods, biopreservation, and active packaging), none of which individually compromises the product’s quality, but together create an impenetrable barrier to microorganisms [5]. Figure 1 shows the physicochemical changes occurring in meat during storage and innovative methods to counteract them.

The aim of this review article is to provide a comprehensive and in-depth analysis of the latest scientific advances in the field of innovative methods for the preservation of meat and meat products. The article discusses in detail the mechanisms of molecular and microbiological non-thermal physical technologies, with particular emphasis on emerging methods (cold plasma, pulsed light, rotating and oscillating magnetic fields). The potential for replacing synthetic preservatives with biological biopreservation methods (protective cultures, bacteriocins, and nanoencapsulated plant extracts) is assessed. Furthermore, breakthroughs in packaging engineering (active biopolymer films and freshness indicators) are presented, and the role of the digital transformation of Industry 4.0 (AI, ML, IoT, HSI) in predictive quality management and real-time cold chain monitoring are demonstrated.

## 2. Review Article Methodology

A literature review was conducted in February 2026 and updated in August 2026 (to include recently published research results) using key international databases indexing peer-reviewed publications: Scopus, Web of Science, PubMed, and ScienceDirect. The search covered publications from the past eleven years (2016–2026), capturing the latest trends in meat preservation technology. Complex logical queries were constructed using Boolean operators (AND, OR). Basic set words key words in the English language included the following combinations: (“meat preservation” OR “meat shelf-life”) AND (“non-thermal technologies” OR “high pressure processing” OR “HPP” OR “ultrasound” OR “cold plasma” OR “pulsed light” OR “magnetic field” OR “biopreservation” OR “clean label” OR “active packaging” OR “intelligent packaging” OR “smart freshness indicators” OR “artificial intelligence” OR “machine learning” OR “IoT”). A total of over 800 articles were identified. After removing duplicates and initial abstract screening, 146 peer-reviewed research studies, review articles, and meta-analyses were selected for full analysis. The selection process for the identified items was multi-stage:

1. Data collection and duplicate removal. All identified items were exported to bibliographic formats (RIS/CSV) and imported into bibliography management software. Zotero (version 9.0.5) was used to organize scholarly sources and manage citations. Duplicates were eliminated by comparing unique DOI (Digital Object Identifier) numbers between databases and by textual normalization of article titles (removing punctuation, converting letters to lowercase, and removing unnecessary spaces).
2. Initial screening. The reviewer assessed the articles’ compliance with the inclusion criteria based on their titles and abstracts.
3. Full-text analysis. Articles qualified for the next stage underwent a detailed full-text analysis for methodological completeness and substantive contribution to the objectives of this study.

The search was limited to articles published in English. This process was supported by manual review of references in key review articles to identify additional items. Narrative review articles review was included selectively—where appropriate—to provide contextual background and explain biological mechanisms. Papers meeting the following conditions were qualified for the final literature review:

1. Chronological scope
  - Publications published in 2016–2026.
  - In the case of issues related to advanced packaging systems (nanotechnology, halochromic indicators) and Industry 4.0 technologies (Internet of Things, Artificial Intelligence, Blockchain), priority was given to the latest literature published after 2019.
2. Thematic scope (subject of research)
  - Research matrix. Studies in which the main object of analysis was fresh meat (pork, beef, poultry, lamb) or processed meat.
  - Fixation technologies. Works evaluating the effectiveness of non-thermal technologies (HPP, HIU, ACP, PL, PEF—Pulsed Electric Fields) in the context of pathogen eradication and shelf life extension while monitoring physicochemical changes in the matrix.
  - Biopreservation. Articles analyzing the use of protective cultures, bacteriophages, phytoextracts and essential oils, including research on their nanoencapsulation and impact on the sensory profile of meat.
  - IoT sensor networks and machine learning algorithms for cold chain management.
3. Type of publication and quality of sources
  - Peer-reviewed original articles with clear research methodology and measurable results (e.g., log units of bacterial reduction, TVB-N concentration, color parameters).
  - Comprehensive review articles and meta-analyses published in high impact factor (IF) journals.
  - Publications indexed in international, verifiable databases: Scopus, Web of Science, ScienceDirect, PubMed.
4. Linguistic criterion
  - Articles published entirely in English to maintain international standards and ensure precise verification of technological terminology.

Publications that met any of the following criteria were ultimately excluded from the analysis:

1. Inappropriate subject and scope of research
  - Inappropriate food matrix. Studies conducted exclusively on other food products (e.g., fish, seafood, dairy products, fruits, and vegetables), unless the results directly referred to analogous mechanisms in meat tissue.
  - No application in a real matrix (in vitro). Work limited solely to preliminary in vitro studies (e.g., disk diffusion tests of essential oils in Petri dishes) without testing their efficacy directly on fresh meat or in the packaging system.
  - Traditional Thermal Technologies. Articles focusing solely on conventional heat preservation methods (pasteurization, sterilization, and freezing) without examining non-thermal solutions, biopreservation, or next-generation packaging systems.
2. Time and technological constraints
  - Publications published before 2015 (excluding works of key historical or definitional importance).
  - Articles describing packaging or IT solutions that have become technologically outdated in the context of contemporary requirements of Industry 4.0 and the Clean concept Label.
3. Type of publication and methodological quality
  - Non-peer-reviewed materials, abstracts from conference proceedings, scientific communications, popular science articles, industry columns and pre-prints that have not successfully passed the peer-review process.
  - Publications from journals of questionable scientific reputation (so-called “predator journals”).
  - Studies showing serious methodological deficiencies, such as lack of experimental repetitions, lack of statistical analysis of results, lack of control groups or imprecise description of process parameters (e.g., failure to provide pressure in HPP, radiation dose in PL or exact chemical composition of the nanocomposite).
4. Formal restrictions and availability
  - Works published in languages other than English.
  - Articles for which the full text of the publication was not available, which prevented full verification of the methodology and results.

## 3. Non-Thermal Fixation Technologies

Non-thermal preservation methods rely on the delivery of physical energy (mechanical, electromagnetic, and electrical) directly to the food matrix without raising its temperature to a level that causes denaturation of heat-labile proteins and loss of nutritional value. Table 1 compares selected leading non-thermal meat preservation technologies.

### 3.1. High Hydrostatic Pressure (HPP)

High Pressure Processing (HPP) involves subjecting the packaged product to pressure in the range of 300–600 MPa in an isostatic liquid medium for a period ranging from several dozen seconds to several minutes. Pascal’s Principle ensures that the pressure is distributed instantaneously and evenly throughout the product, regardless of its shape and mass. The main mechanism of microorganism inactivation in HPP technology is the disruption of the cell membrane, change in its permeability, crystallization of membrane lipids, and inactivation of intracellular enzymes crucial for DNA replication [25]. The application of 600 MPa pressure to ready-to-eat (RTE) meat products allows for the reduction in the *Listeria monocytogenes* population by >5 log_10_ cfu/g, which eliminates the risk of secondary post-process infection [26,27]. However, the application of HPP to fresh, raw red meat has certain limitations. Pressures above 300 MPa induce myoglobin denaturation and the release of the heme group, which leads to an unfavorable color change (the so-called “cooked effect”—light gray color) and induces lipid peroxidation involving free iron [28]. New research focuses on reducing the applied pressure to 200–250 MPa and combining it with the addition of natural antioxidants (e.g., olive leaf extract) or vacuum packaging, which minimizes oxidative changes while maintaining microbiological safety [29,30,31]. Due to the risk of lipid oxidation and fading of raw meat, HPP is often combined with natural antioxidants. In the analyzed studies, HPP was typically applied at 300–600 MPa for 1–5 min, depending on the meat matrix and target microorganisms. Pressures of 600 MPa enabled a reduction in *Listeria monocytogenes* by >5 log_10_ cfu/g, confirming high efficacy in ready-to-eat (RTE) products. However, pressures exceeding 300 MPa induced myoglobin denaturation and lipid oxidation, resulting in the characteristic “cooked effect” and increased malondialdehyde (MDA) formation [27,32]. Recent studies therefore emphasize the use of lower pressures (200–250 MPa) combined with natural antioxidants (e.g., olive leaf extract) or vacuum packaging to minimize oxidative changes while maintaining microbiological safety [31].

The study by Kumar et al. (2026) [32] analyzes the impact of high hydrostatic pressure (HPP, in the 100–600 MPa range) applied under both refrigerated and elevated-temperature conditions to various meat raw materials (beef, pork, poultry) as well as cured and processed meat products. The research evaluated color components within the CIEL*a*b* system, the chemical state of myoglobin, structural changes in fibrous proteins (light scattering), and lipid oxidative stability. HPP treatment at levels exceeding 200–400 MPa resulted in undesirable lightening (increased L* value) and a loss of redness (decreased a* value) in fresh red meat, driven by protein aggregation and myoglobin oxidation—creating a visual appearance similar to that of cooked meat. This mechanism differs from thermal processing, as HPP primarily disrupts non-covalent bonds. Cured meat products and poultry exhibit significantly greater color stability under pressure. The authors emphasize the need to avoid pressures exceeding 400 MPa for fresh red meat and suggest the implementation of “clean-label” approaches (such as the use of natural antioxidants, active packaging, or hybrid systems combining HPP with cold plasma or sous-vide processing).

Chmiel et al. 2025 [33], an experiment was conducted on raw culinary beef (*m. semimembranosus*) packaged using a vacuum skin-pack system and subjected to High-Pressure Processing (HPP) at 300, 400, and 500 MPa for 5 min at 5 °C. The samples were stored under refrigerated conditions (0–4 °C) for 56 days, with analyses performed at 9 time points. The study evaluated pH, CIEL*a*b* color parameters (including total color difference), the degree of lipid oxidation (TBARS index), and the profile of 9 essential free amino acids. The application of HPP—particularly at 500 MPa—effectively stabilized the beef’s pH at 5.6–5.7 and inhibited the growth of acidifying microflora; however, it also caused a noticeable lightening of the meat and a more than threefold increase in the TBARS index (reaching 1.9 mg MDA/kg by day 56). High-pressure treatment reduced the concentration of most free amino acids compared to the control group (with the exception of leucine, phenylalanine, and lysine) and slowed their release during the aging process. The authors conclude that to minimize negative quality changes—such as lipid oxidation, color alteration, and the loss of amino acids—while maintaining the microbiological stability of culinary beef, the use of lower pressure ranges (300–400 MPa) is optimal.

### 3.2. High Intensity Ultrasound (HIU)

Ultrasound with high power density (>1 W/cm^2^) and low frequency (20–100 kHz) operates on the principle of transient acoustic cavitation. The passage of an ultrasonic wave through a liquid medium creates cycles of stretching and compression, generating microscopic gas bubbles that grow to a critical size and then violently implode [34]. The implosion of bubbles causes highly localized physicochemical phenomena:

- temperature jumps up to 5000 K and pressure up to 100 MPa on a microscale,
- formation of micro-fluid streams penetrating muscle tissue at a speed of several hundred m/s,
- sonolysis of water with generation of free radicals (H^•^, OH^•^) [18].

The use of HIU in meat processing is multifunctional. Ultrasound waves have been widely used to modify the technological properties of meat. The phenomenon of acoustic cavitation accelerates, among other things, the diffusion of salts during curing [35]. Ultrasound is effective in surface microbiological decontamination [36], affects the structure and oxidation of proteins [37], and also plays a crucial role in the processes of inducing the desired tenderness of the meat matrix [18,19]. Ultrasound waves cause sonoporation of cell membranes of spoilage microorganisms, accelerate mass transfer during salting and curing (reducing the process time by up to 30–50%), and disrupt myofibrillar structures, improving meat tenderness and water absorption. To limit the negative impact of the generated hydroxyl radicals on fat oxidation, recent trends suggest combining HIU with natural free radical scavengers (e.g., green tea polyphenols) [38]. HIU treatments were conducted at 20–40 kHz, with power densities exceeding 1 W/cm^2^ and exposure times of 5–15 min. Cavitation phenomena accelerated salt diffusion during curing, reducing processing time by 30–50%, and improved tenderness by disrupting myofibrillar structures (decrease in shear force by 10–20%). Surface decontamination typically resulted in microbial reductions of 1–2 log units. However, the generation of hydroxyl radicals (OH^•^) increased lipid oxidation, reflected by elevated MDA levels, which can be mitigated by combining HIU with natural antioxidants [35,39].

The study by Graciano et al. (2025) [40] analyzed the application of ultrasound (wave frequency, intensity, and exposure time) to improve beef tenderness. The parameters evaluated primarily included Warner–Bratzler shear force, muscle structure, proteolytic activity, water-holding capacity, color, and oxidative changes. The results indicate that ultrasound affects muscle tissue through acoustic cavitation, leading to the fragmentation of muscle fibers and the intensification of enzymatic proteolysis associated with the activation of the calpain system. These changes result in reduced shear force and improved meat tenderness. A significant reduction in shear force values and beneficial microstructural changes in the muscle were also observed. At the same time, the authors emphasize that the technology’s effectiveness depends on the proper selection of process parameters. Excessive intensity or prolonged treatment time can lead to increased lipid and protein oxidation, potentially negatively affecting product quality. Consequently, ultrasound is considered a promising method for improving beef tenderness, though it requires further optimization prior to widespread industrial implementation.

In a study by Son et al. (2024) [39], the impact of high-intensity ultrasound (HIU: 2400 W, 36.2 kHz, 90 min) combined with a wet-dry aging process on the quality of pork loin was evaluated. The samples underwent wet aging for 7, 14, or 21 days, followed by dry aging for 3 or 7 days. The parameters analyzed included color (L*, a*, b*), pH, water-holding capacity, cooking loss, Warner–Bratzler shear force (WBSF), and the extent of lipid oxidation, measured via the TBARS assay. The results demonstrated that the application of HIU significantly improved meat tenderness by reducing WBSF values in most of the aging variants tested. This effect was attributed to alterations in the myofibrillar structure and an intensification of muscle protein degradation. Furthermore, an increase in pH and improvements in water-retention parameters were observed during the aging process, which may positively influence the juiciness of the product. Conversely, the ultrasonic treatment led to higher TBARS values, indicating an acceleration of lipid oxidation. This suggests that the improvement in meat tenderness was partly linked to a decline in oxidative stability. The authors concluded that the most favorable outcomes were achieved by combining HIU with 14 days of wet aging followed by 3–7 days of dry aging, which provided an optimal balance between enhanced texture and minimized oxidative changes.

### 3.3. Atmospheric Pressure Cold Plasma (ACP)

Atmospheric pressure cold plasma (ACP) is considered one of the most promising innovations of the last decade. Plasma, called the fourth state of matter, is created by ionization of a gas (e.g., air, argon, helium, or gas mixtures) in a strong electric field [41]. In cold plasma, the temperature of electrons is high, while the temperature of heavy molecules (ions, neutral atoms) remains close to ambient temperature, which prevents thermal damage to the tissue. The ACP decontamination mechanism is based on the generation of reactive oxygen and nitrogen species (RONS), such as ozone (O_3_), singlet oxygen (^1^O_2_), hydroxyl radicals (OH^•^), nitric oxide (NO^•^), and UV radiation [42]. RONS cause powerful oxidative stress on the surface of bacterial cells, leading to damage to lipid membranes, destruction of cell wall proteins, and DNA erosion (etching effect). Dielectric Barrier Discharge (DBD) plasma treatment directly inside the sealed poultry package reduces the *Salmonella* population *Typhimurium* and *Campylobacter jejuni* by >3.5 log_10_ in less than 3 min [20]. The main technological challenge is the fact that the strong oxidizing properties of RONS can initiate the oxidation of lipids and myoglobin. Therefore, recent studies emphasize the precise optimization of the working gas composition and exposure time in order to spare the sensory profile [21,22,43]. Dielectric Barrier Discharge (DBD) plasma systems operated at 60–90 kV for 1–3 min, using air, argon, or helium as working gases. ACP generated reactive oxygen and nitrogen species (RONS), including O_3_, ^1^O_2_, OH^•^, and NO^•^, which caused oxidative damage to microbial membranes and DNA. Treatment inside sealed poultry packages reduced *Salmonella Typhimurium* and *Campylobacter jejuni* by >3.5 log_10_ within 3 min. Quality assessments focused on color, lipid oxidation (MDA), and TVB-N, as prolonged exposure may induce undesirable oxidative changes [20,42].

The study by Abdel-Naeem et al. (2022) [44] aimed to evaluate the efficacy of low-temperature plasma, generated via Dielectric Barrier Discharge (DBD), for the decontamination of chicken breast fillets. The process utilized two different gases (argon and oxygen), a voltage of 20 kV, and exposure durations of 3 and 5 min. The researchers assessed microbiological quality, physicochemical parameters (pH, TVBN, TBA, color, shear force, and cooking loss), sensory attributes, and microstructural changes (using scanning electron microscopy). The results demonstrated a significant reduction in microbial counts across all treatment variants, with plasma generated in an argon atmosphere proving more effective than that generated in oxygen. Furthermore, the treatment did not negatively affect lipid stability (TBA), the degree of protein degradation (TVBN), or cooking loss. The treatment led to a decrease in both pH and the shear force of the meat, indicating an improvement in the product’s tenderness. Microstructural analysis confirmed the degradation of muscle fibers and connective tissue—particularly in samples treated with argon—which correlated with higher sensory scores for tenderness. The authors concluded that the application of DBD plasma, especially when using argon for 3 min, represents an effective and safe method for decontaminating poultry meat without compromising its technological or sensory quality.

Research by Csadek et al. (2023) [45] focused on evaluating the efficacy of cold atmospheric plasma (CAP) in reducing microbial contamination on the surface of ready-to-eat (RTE) meat products, such as cooked ham, sausages, roasted veal, and pâté. Samples were exposed to plasma generated in air for 2–5 min at two different power levels. The study assessed color changes (L*, a*, b*, ΔE) as well as the reduction in populations of *Listeria monocytogenes*, *Listeria innocua*, and *Escherichia coli*. The results indicated that CAP exhibits antimicrobial activity, particularly against Gram-negative bacteria. The reduction in *E. coli* counts reached up to 3 log CFU/g, while the efficacy against *Listeria* species was lower, peaking at approximately 1.5 log CFU/g. At the same time, the treatment had a minimal impact on the visual properties of the products. A slight decrease in the a* parameter value was observed; however, the total color change (ΔE) did not exceed the threshold considered significant from a consumer’s perspective. The authors emphasized that the process’s efficacy varied depending on the type of product, and that plasma could serve as an additional tool for mitigating the risk of secondary contamination in RTE products without significantly compromising their sensory quality.

In a study by Hui et al. (2023) [46], the impact of cold atmospheric plasma (CAP)-assisted curing on the quality and safety of beef—roasted using both a traditional method (over charcoal) and superheated steam—was evaluated. Color parameters, aroma profile, volatile aroma compound composition, and the content of heterocyclic amines (HAs)—compounds considered potentially mutagenic and carcinogenic—were analyzed. The results demonstrated that the application of CAP enhanced the red color intensity of the meat, particularly with superheated steam treatment, where redness increased by approximately 46%. At the same time, the process inhibited the formation of undesirable lipid oxidation products responsible for aroma deterioration, especially in samples roasted in superheated steam. A significant finding was also that CAP did not increase the content of heterocyclic amines, thereby not raising the health risks associated with heat-treating meat. The authors highlighted the synergistic effect of plasma and superheated steam roasting, which allows for the simultaneous improvement of the product’s sensory characteristics and the preservation of its safety.

### 3.4. Pulsed Light Beam (PL) and Pulsed Electric Field (PEF)

Pulsed light beam technology (PL) uses short-duration (100 μs–1 ms), highly energetic flashes of light with a broad electromagnetic spectrum (200–1100 nm), rich in the UV-C radiation range [47]. The biocidal mechanism of PL is photochemical and photothermal. UV-C radiation induces the formation of pyrimidine dimers in the DNA/RNA structure of microorganisms, which prevents their replication, while impulsive light energy causes local overheating and disruption of cell walls [48]. PL is excellent for surface decontamination of poultry carcasses, salmon flaps, and sliced meat products. Exposure of ham slices to several PL flashes with a total energy of 3–5 J/cm^2^ reduces the *L. monocytogenes* microflora without raising the product temperature above 4° C [23]. In parallel, the technology of pulsed electric field (PEF), which involves treating food with short high-voltage pulses (1–10 kV/cm). PEF induces the phenomenon of electroporation—the formation of pores in the lipid membrane of cells, which leads to the escape of their intracellular contents and death. In muscles, PEF also accelerates maturation processes and facilitates the penetration of brines [24]. PL systems delivered 100 μs–1 ms pulses with energies of 3–5 J/cm^2^ across the 200–1100 nm spectrum. Application of several pulses to ham slices reduced *L. monocytogenes* without increasing product temperature above 4 °C. Typical microbial reductions ranged from 1–3 log units, although uneven surfaces may exhibit the “shadowing effect.” Quality parameters included color, texture, and lipid oxidation [23,24,47]. PEF treatments used electric field strengths of 1–3 kV/cm, pulse durations of 20–80 μs, and 50–200 pulses. Electroporation enhanced brine penetration (20–30% faster diffusion) and improved tenderness (10–15% reduction in shear force). Microbial reductions of 1–2 log units were observed, with conductivity of the medium being a critical factor for process efficiency [24].

The study by Söbeli et al. (2021) [49] aimed to evaluate the impact of pulsed UV-C light on the microbiological quality and selected physicochemical properties of raw beef steaks. Samples were exposed to UV-C doses of 0.525, 1.05, 2.1, and 4.2 J/cm^2^. The researchers assessed the proximate composition of the meat (moisture, protein, fat, and ash content), pH, color parameters (CIE L*a*b* system), aerobic mesophilic bacterial counts, and the profile of volatile aromatic compounds. The results indicated that UV-C treatment did not significantly affect the chemical composition or pH of the meat. However, an increase in the radiation dose led to a decrease in the a* and b* color parameters, reflecting a reduction in the intensity of the meat’s red and yellow hues. The highest antimicrobial efficacy was observed at a dose of 4.2 J/cm^2^, resulting in a reduction in the total aerobic mesophilic bacterial count by approximately 3.5 log CFU/g. Changes in the profile of volatile compounds were also noted, primarily linked to an intensification of lipid oxidation processes. The authors concluded that pulsed UV-C technology can effectively enhance the microbiological safety of beef while preserving its fundamental quality characteristics.

In a study by Cassar et al. (2021) [50], the efficacy of pulsed ultraviolet (PUV) light—applied via a moving conveyor belt—was evaluated for the decontamination of poultry meat cuts. Breast fillets, skinless thighs, and skin-on thighs were analyzed after being exposed to energy doses of 5, 10, 20, and 30 J/cm^2^. The study evaluated the reduction in *Escherichia coli* counts, the degree of lipid oxidation (TBARS), color parameters (L*, a*, b*), and changes in the surface temperature of the meat. The results showed that antimicrobial efficacy increased with higher radiation doses. The greatest reduction in *E. coli* counts was achieved at 30 J/cm^2^, with higher efficacy observed on skinless surfaces compared to skin-on meat. At the same time, the treatment had no significant impact on the degree of lipid oxidation or the color parameters of the meat during a five-day storage period. An increase in the surface temperature of the samples was observed as the energy dose increased. The authors concluded that pulsed UV (PUV) technology could be an effective method for reducing microbial contamination in poultry meat without causing significant changes to the product’s quality.

Research by McConnell et al. (2025) [51] evaluated the efficacy of a novel, double-sided photonic decontamination system that uses pulsed light to reduce microbial contamination on poultry meat cuts. Chicken wings and tenderloins were analyzed; after being artificially contaminated, they were subjected to pulsed light treatment—either alone or in combination with immersion in water or a peracetic acid (PAA) solution. The study assessed the counts of aerobic plate count (APC) bacteria, *Enterobacteriaceae*, and the pathogens *Salmonella* and *Campylobacter*. The results indicated that the application of pulsed light led to a significant reduction in the counts of indicator bacteria and *Salmonella* on both chicken wings and tenderloins. Microbial reductions ranged from 0.25 to 1.18 log CFU/mL, with the best results achieved when pulsed light was combined with a prior immersion in a peracetic acid (PAA) solution. For *Campylobacter*, the efficacy of the process varied depending on the type of poultry cut. The authors emphasized that photonic decontamination technology could serve as a valuable supplement to existing antimicrobial methods in the poultry industry, particularly as an additional step for limiting the microbial contamination of products.

The study by Baldi et al. (2021) [52] aimed to evaluate the impact of pulsed electric field (PEF) treatment on the technological properties of chicken meat. Samples of the *pectoralis major* muscle were subjected to an electric field with an intensity of 0.6 or 1.2 kV/cm, using between 150 and 600 pulses. The parameters assessed included pH, color (L*, a*, b*), water-holding capacity (drip loss), protein solubility, and the enthalpy of protein denaturation. The results indicated that the PEF parameters applied did not significantly affect the pH of the meat, nor did they alter the degree of denaturation or the functionality of the muscle proteins. Only minor changes in the lightness and yellowness of the meat were observed, with no significant impact on the attributes that determine consumer acceptability. The most notable effect of the technology was an improvement in the meat’s water-holding capacity. PEF treatment reduced drip loss by up to 28.5% compared to control samples; increasing the number of pulses proved more effective in achieving this result than increasing the electric field intensity. The authors conclude that low-energy pulsed electric fields can serve as an effective tool for enhancing the technological properties of poultry meat by reducing water loss during storage, without negatively affecting protein structure or the product’s fundamental quality characteristics.

The study by Bolumar et al. (2022) [53] aimed to optimize pulsed electric field (PEF) parameters to improve the tenderness of the bovine *semimembranosus* muscle. Three electric field strengths (0.25, 0.50, and 1.00 kV/cm), two pulse frequencies (20 and 100 Hz), and three treatment durations (10, 30, and 50 ms) were applied. The study evaluated changes in pH, drip loss, cooking loss, color parameters, and meat tenderness—the latter measured via Warner–Bratzler shear force—after 1 and 14 days of refrigerated storage. The results indicated that no improvement in meat tenderness was observed immediately following the pulsed electric field (PEF) treatment. Beneficial effects emerged only after 14 days of refrigerated storage, with certain treatment variants showing a 5–10% reduction in shear force compared to control samples. The best results were achieved at lower electric field strengths (0.25–0.50 kV/cm), whereas a higher intensity (1.00 kV/cm) provided no additional technological benefits and, in some cases, led to a decline in product quality. Furthermore, PEF treatment had no significant impact on pH, meat color, cooking loss, or drip loss during storage. The authors suggest that the observed improvement in tenderness may be linked to alterations in muscle microstructure and an intensification of proteolytic processes occurring during the meat-aging (maturation) period. They also conclude that the application of low-intensity electric fields may be a promising method for assisting the beef-aging process, without negatively affecting its key quality characteristics.

### 3.5. Rotating and Oscillating Magnetic Field (RMF/OMF)

An innovation in the field of technologies supporting fixation is the use of magnetic fields: oscillating (OMF) and rotating (RMF). Magnetic fields with intensities ranging from a few milli-Tesla to several dozen Tesla affect the electric charges of cell membranes and the orientation of water molecule dipoles [54]. The interaction of RMF and OMF causes disruptions in the transport of ions through membrane channels (especially Ca^2+^ and Na^+^ ions), which leads to changes in the membrane potential of microorganisms and their lysis. Furthermore, studies show that magnetic fields can modify the meat freezing process (Magneto- Assisted Freezing) [55]. The use of OMF during freezing induces the formation of numerous, microscopic water crystallization nuclei. As a result, the ice crystals formed are small and do not damage the cell membranes of muscle tissue, which significantly reduces free leakage (drip loss) during defrosting and allows to maintain optimal juiciness of the meat [56]. The synergistic effect of a rotating magnetic field with a pulsed light beam (RMF-PL) has also been demonstrated. The combined application of these two methods to raw pork allowed for extending its shelf life by 30% while reducing the total energy consumption compared to individual techniques [57].

The study by Kang et al. (2022) [58] evaluated the impact of a pulsed electric field (PEF, 100 Vp/m; 20 kHz) and an oscillating magnetic field (OMF, 10 mT; 10 Hz) on the feasibility of storing beef in a supercooled state—that is, below its freezing point without the formation of ice crystals. Two types of beef with varying fat content—lean beef and beef with a higher fat content—were analyzed. Samples were stored at approximately −4 °C for up to 7 days, with the effectiveness of supercooling compared against conventional refrigeration and freezing. Additionally, computer simulations were conducted to assess temperature distribution and current flow within the meat, accounting for different degrees of fat content. The study evaluated the chemical composition of the meat, its thermophysical and electrical properties, the stability of the supercooled state, color changes (parameter a*), and drip loss (purge) as an indicator of damage to the muscle tissue structure. The results demonstrated that the effectiveness of supercooling depended on the fat content of the meat. For lean beef, the application of PEF alone was sufficient to maintain the supercooled state, whereas for fattier beef, the simultaneous application of both PEF and OMF was required. The analyses revealed that fat, due to its low electrical conductivity, disrupted uniform current flow and temperature distribution, thereby increasing the risk of ice crystallization. The synergistic effect of electric and magnetic fields enabled the effective inhibition of ice nucleation, keeping the meat in a supercooled state for 7 days. Quality assessment of the stored meat revealed that the supercooled samples exhibited significantly lower drip loss compared to frozen samples, as well as better retention of their red color when compared to meat stored under conventional refrigeration. These results indicate that supercooling technology, assisted by pulsed electric fields (PEF) and oscillating magnetic fields (OMF), can extend the shelf life of beef while minimizing the quality degradation typically associated with freezing. The authors consider this method a promising alternative to conventional meat storage techniques, particularly for products that require the preservation of fresh-like characteristics.

## 4. Biopreservation and the “Clean Label” Trend

Biopreservation is defined as the use of natural microorganisms, their metabolic products or declared substances of bio -plant origin to inhibit the development of spoilage and pathogenic microflora [59]. Table 2 summarizes meat biopreservation techniques.

### 4.1. Lactic Acid Bacteria, Bacteriocins, and Bacteriophage Therapy

Protective cultures, composed of selected strains of lactic acid bacteria (LAB, e.g., *Lactilactobacillus sakai*, *Latilactobacillus curvatus*, *Carnobacterium maltaromaticum*), are introduced into meat products to dominate the microenvironment. LAB compete with pathogens for nutrients and adhesion sites and produce organic acids (lactic, and acetic), lowering the pH of the environment [64]. The use of protective cultures is one of the main pillars of biopreservation in the era of the clean label trend [65]. A modern extension of the LAB use concept is the use of postbiotics, i.e., cell-free supernatants (CFS) containing a unique cocktail of secondary metabolites, enzymes, organic acids, and peptides. The application of metabolites alone, bypassing live bacterial cells, completely eliminates the risk of uncontrolled fermentation and excessive acidification of the product during long-term refrigerated storage, which is particularly desirable in the case of fresh meat packaging [13]. A particularly important tool are bacteriocins produced by LAB—highly specific antimicrobial peptides (e.g., nisin, pediocin PA-1, and enterocins). Bacteriocins are incorporated into the cell membrane of target Gram-positive bacteria, creating permeable pores, which leads to ATP drainage and cell death [66]. The use of isolated, defined bacteriocins allows for precise eradication *L. monocytogenes* in cured meats without imparting a sour fermented flavor to the product. Recent research emphasizes the need to incorporate bacteriocins into so-called hurdle technology systems. Because bacteriocins exhibit limited effectiveness against Gram-negative bacteria (those with a protective outer membrane), they are combined with mild physical methods, such as high-pressure food preservation (HPP), or essential oils. This synergistic effect destabilizes the cell membrane of pathogens, sensitizing them to antimicrobial peptides and preventing the development of resistant strains [14]. Protective cultures were applied at 10^6^–10^8^ CFU/g (Colony-Forming Unit per gram), lowering pH by 0.3–0.6 units and reducing *L. monocytogenes* by 1–2 log units. Postbiotics (cell-free supernatants) prevented excessive acidification during refrigerated storage, making them suitable for fresh meat packaging [65,67]. Bacteriocins such as nisin, pediocin PA-1, and enterocins were used at 200–1000 IU/g (International Units per gram), achieving 2–4 log reductions in Gram-positive pathogens without affecting sensory attributes. Their limited activity against Gram-negative bacteria necessitates synergistic combinations with HPP or essential oils [67].

The study by Ying et al. (2025) [67] aimed to evaluate the efficacy of bacteriocin XJS01 as a natural preservative for beef stored at 4 °C for 12 days. Meat samples were treated with two concentrations of the bacteriocin (1 × MIC and 2 × MIC), followed by an analysis of quality changes and the metabolomic profile. The parameters assessed included microbiological counts (mesophilic bacteria and *E. coli* O157:H7), pH, total volatile basic nitrogen (TVB-N), thiobarbituric acid reactive substances (TBARS), color parameters (L*, a*, b*), and sensory attributes. The results demonstrated that XJS01 effectively inhibited microbial growth and slowed down meat spoilage processes. Samples treated with the bacteriocin exhibited lower pH, TVB-N, and TBARS values, as well as better retention of color and sensory acceptability compared to the control group. Metabolomic analysis indicated that the mechanism of action of XJS01 involved the modification of metabolic pathways in spoilage-causing bacteria—specifically those related to the metabolism of amino acids, sugars, and phospholipids. Furthermore, a reduction in the formation of dipeptides—byproducts of protein degradation—was observed. The authors concluded that XJS01 could serve as an effective biological preservation method for beef, while also facilitating the identification of metabolites that act as markers for meat freshness.

Study by Qi et al. (2026) [68] focused on the isolation and characterization of the *Lactiplantibacillus plantarum* BL25 strain and the mixture of bacteriocins it produces, designated as QBL25—which includes plantaricin E and a newly identified bacteriocin, QBL25-1. The study evaluated the antimicrobial properties, physicochemical stability, and antioxidant activity of this preparation, as well as its efficacy in preserving beef stored under refrigeration for 8 days. Parameters monitored included microbial counts, pH, color, TBARS, TVB-N, and sensory characteristics of the meat. The results demonstrated a broad antimicrobial spectrum against both Gram-positive and Gram-negative bacteria, along with high stability across a wide range of temperatures and pH levels. The mechanism of action of QBL25 involves damaging the cell membrane of *Enterobacter cloacae* and disrupting its transport functions. In the storage experiment, the addition of QBL25 inhibited the growth of spoilage-causing bacteria, slowed lipid oxidation, limited the increase in TVB-N values, and helped maintain the red color and sensory qualities of the beef. The authors concluded that both the BL25 strain and its bacteriocins hold significant potential as natural preservatives for the meat industry.

The article by Rahman et al. (2026) [69] focuses on the application of lactic acid bacteria (LAB) and the antimicrobial substances they produce—primarily bacteriocins and postbiotics—for the biological preservation of meat and meat products. The authors analyzed the potential for using starter cultures, protective cultures, postbiotics, and purified bacteriocins in various meat production systems. Particular attention was paid to the impact of these methods on the microbiological safety, shelf life, and the physicochemical and sensory quality of the products. The cited findings indicate that lactic acid bacteria (LAB) and their metabolites effectively inhibit the growth of pathogenic bacteria and spoilage-causing microorganisms, such as *Listeria monocytogenes*, *Salmonella* spp., *Escherichia coli*, and *Staphylococcus aureus*. These studies observed a decrease in pH, a slowing of lipid and protein oxidation, a reduction in total volatile basic nitrogen (TVB-N) values, and the improvement or preservation of desirable sensory attributes, such as color, texture, and flavor. Especially promising results were obtained when using bacteriocins in combination with other preservation methods, in line with the “hurdle technology” concept. The authors emphasize that postbiotics and bacteriocins can serve as an effective alternative to synthetic preservatives, addressing the growing consumer demand for “clean label” food products. At the same time, they point to the need for further research regarding the standardization of production methods, the safety assessment of microbial strains, and the efficacy of implementing these technologies on an industrial scale across various meat products.

Another bio- weapon in the fight against pathogens is bacteriophage therapy. Bacteriophages are lytic viruses that specifically infect and destroy specific species or strains of bacteria, while being completely harmless to humans and eukaryotic cells. Commercial phage preparations (e.g., those directed against *Listeria monocytogenes* or *Salmonella enterica*) are applied by spraying on the meat surface. Some studies confirm their high effectiveness without affecting the physicochemical parameters and sensory characteristics of the raw material [12]. Currently, the evolution of classical phage therapy is the use of endolysins Phage enzymes—isolated lytic enzymes by which viruses degrade the host cell wall in the final phase of the infection cycle. Endolysins, unlike whole bacteriophages, attack the peptidoglycan structure from outside the cell (which is highly effective, especially against Gram-positive bacteria), acting almost immediately. Furthermore, their isolated use bypasses bacterial defense mechanisms (e.g., CRISPR—Cas systems—Clustered Regularly Interspaced Short Palindromic Repeats—CRISPR-associated proteins), significantly reducing the likelihood of pathogens developing cross-resistance in meat processing plants [16]. Phage preparations were applied at 10^7^–10^9^ PFU/cm^2^ (Plaque-Forming Units per square centimeter), achieving 1–3 log reductions in *Listeria* and *Salmonella* without altering physicochemical properties. Endolysins acted rapidly against Gram-positive bacteria and bypassed bacterial CRISPR-Cas defense systems, reducing the risk of resistance development [16].

The study by Cui et al. (2026) [70] aimed to isolate and characterize the lytic bacteriophage vB_AbaP_P4, intended for controlling multidrug-resistant *Acinetobacter baumannii* (MDR-AB) strains, and to evaluate its potential as a biological preservative in food products. The research encompassed an analysis of the phage’s biological and genomic properties, an assessment of its antibacterial activity against planktonic cells and biofilms, and application tests using food models, specifically chicken meat. The study evaluated the phage’s stability under various pH and temperature conditions, its host range (lytic spectrum), growth kinetics, efficacy in biofilm removal, and its ability to reduce bacterial counts in food. The results demonstrated that phage P4 exhibits high stability across a broad range of pH (3–11) and temperatures (30–60 °C). Furthermore, genomic analysis revealed no genes associated with antimicrobial resistance, virulence factors, or lysogeny-related elements, confirming its biological safety. In vitro experiments showed effective inhibition of bacterial growth and significant anti-biofilm activity; the phage reduced biofilm formation by approximately 17% and decreased the number of biofilm-associated cells by up to 6 log units. In the case of mature biofilms, the phage achieved the removal of up to 91% of the biomass and a 3.5 log reduction in the number of viable cells. In experiments conducted on artificially contaminated chicken meat, the application of the phage resulted in a 1.6 log CFU/g reduction in the count of multidrug-resistant *Acinetobacter baumannii* (MDR-AB) over 24 h of storage. These findings suggest that phage P4 is a promising tool for the biological control of microbial contamination in food, particularly regarding multidrug-resistant and biofilm-forming strains. However, the authors emphasize the need for further research under industrial conditions and on naturally contaminated food products.

A study by Huang et al. (2025) [71] focused on the isolation and characterization of the bacteriophage vB_EcoM_SD350, which exhibits activity against avian pathogenic *Escherichia coli* (APEC) strains, and evaluated its potential as a biocontrol agent for meat. The research included an analysis of the phage’s biological, genomic, and antimicrobial properties, an assessment of its ability to inhibit bacterial growth and biofilm formation, and application tests on chicken and beef stored for 72 h at 4 °C. The study evaluated the host range (lytic spectrum), stability under various environmental conditions, replication kinetics, genomic composition, and efficacy in reducing bacterial counts in food. The results demonstrated that phage vB_EcoM_SD350 possesses a broad host range, lysing 64% of the *E. coli* strains tested. It exhibited high stability across a pH range of 3–13 and temperatures of 40–60 °C, and genomic analysis confirmed the absence of toxin genes, antibiotic resistance genes, and elements associated with the lysogenic cycle. The phage effectively inhibited bacterial growth and significantly reduced biofilm formation by the host strain. Application studies demonstrated the phage’s efficacy in reducing *E. coli* counts on meat surfaces. In chicken, the reduction in bacterial numbers ranged from 0.56 to 2.88 log CFU/g, while in beef, it ranged from 0.43 to 3.98 log CFU/g, with the highest efficacy observed at the maximum phage concentration (10^8^ PFU/mL). Furthermore, the phage titer remained stable throughout the entire storage period. The authors conclude that vB_EcoM_SD350 could serve as an effective and safe tool for the bioconservation of meat, as well as an alternative to conventional methods for controlling bacterial contamination in products of animal origin.

### 4.2. Plant Extracts, Essential Oils and Nanoencapsulation

Extracts from herbs, spices, and by-products of the fruit and vegetable industry (e.g., grape seeds, citrus peels, olive leaves) are a rich source of natural bioactive substances. They contain phenolic compounds (rosmarinic acid, gallic acid, and catechins) and monoterpenes (thymol, carvacrol, and eugenol), which are characterized by strong antioxidant and antibacterial effects [63,72,73]. These compounds destroy the integrity of bacterial cell membranes and neutralize free radicals responsible for fat oxidation. In recent years, particular emphasis has been placed on the principles of a circular economy, which is driving the extraction of polyphenols from fruit pomace, pomegranate peels, and avocado seeds, which until recently were considered useless waste. Furthermore, to fully comply with clean label requirements, so-called “clean label” methods are now used to isolate these compounds. Green extraction technologies (e.g., supercritical CO_2_ extraction or ultrasound-assisted extraction) that completely eliminate the need for toxic organic solvents, guaranteeing the highest purity of the obtained preparations [17]. Newer molecular studies also show that, in addition to direct cell lysis, phytochemicals (such as cinnamic aldehyde) exhibit strong anti-quorum activity sensing (anti-QS). This mechanism involves disrupting the chemical pathways of interbacterial communication, which effectively paralyzes the ability of meat-spoiling microorganisms (e.g., *Pseudomonas* spp.) to create three-dimensional, detergent-resistant biofilms on the surface of raw materials and processing devices [74,75,76]. The main technological problem related to the direct use of essential oils in meat is their high volatility, poor solubility in water, susceptibility to oxidation and photodegradation, and above all, intense herbal aroma, which in therapeutic doses significantly reduces the sensory acceptability of the product [77].

The study by Belloumi et al. (2025) [78] aimed to evaluate the preservative potential of individual essential oils and their synergistic blends—derived from six species of medicinal and culinary plants (*Thymbra capitata*, *Illicium verum*, *Myristica fragrans*, *Coriandrum sativum*, *Piper nigrum*, and *Carum carvi*)—for extending the shelf life of poultry meat. The research encompassed an analysis of the chemical composition of the oils, an assessment of their antioxidant and antimicrobial activities, the determination of synergistic effects, an evaluation of cytotoxicity, and an investigation into the impact of selected combinations on the sensory quality and shelf life of marinated chicken meat stored at 4 °C. The parameters assessed included free-radical scavenging capacity (DPPH), ferric-reducing power (EC_50_), minimum inhibitory concentrations (MIC) against microorganisms, synergy indices (AFC and FIC), cytotoxicity toward RAW 264.7 cells, sensory attributes of the meat (color, odor, taste, juiciness, tenderness, and overall acceptability), pH changes, and microbial growth during storage. Furthermore, the product’s shelf life was estimated using kinetic models based on the Arrhenius equation. The results demonstrated that the essential oil combinations exhibited significantly stronger biological activity than their individual components. The highest antioxidant activity was observed for the *T. capitata–I. verum* combination, while the *T. capitata–P. nigrum* mixture displayed particularly favorable antimicrobial properties. *P. nigrum* and *I. verum–M. fragrans*, for which a significant reduction in MIC values was observed compared to the oils used individually. Many of the analyzed combinations demonstrated a synergistic effect in terms of both antioxidant and antimicrobial activity. Storage studies revealed that the addition of selected oil blends effectively inhibited microbial growth and stabilized the pH of the meat. The best results were obtained with the *T. capitata–P. nigrum* combination, which extended the microbiological shelf life of the meat from 6 days (in the control group) to 14 days, and with the *I. verum–M. fragrans* blend, which extended it to 12 days. Furthermore, both combinations maintained high sensory acceptability and exhibited relatively low cytotoxicity. The authors conclude that carefully selected essential oil blends can serve as an effective alternative to synthetic preservatives used in the meat industry. The *T. capitata–P. nigrum* and *I. verum–M. fragrans* combinations proved particularly promising, as they combined high antioxidant and antimicrobial activity with favorable sensory properties and a significant extension of the refrigerated shelf life of poultry meat.

Nanoencapsulation is a solution to this problem. Encapsulating active substances in nanocarriers (nanoliposomes, nanoemulsions, solid lipid particles, complexes with cyclodextrins) allows for their protection from external factors, masking the intense flavor, and ensuring controlled, gradual release during meat storage [73]. A practical example of the effectiveness of this technology is the use of a thyme oil nanoemulsion encapsulated in an alginate matrix and added to raw ground beef. This solution allowed for a 60% reduction in the essential oil dose, guaranteeing the maintenance of the original antimicrobial efficacy while eliminating sensory defects [79]. Among the latest nanotechnology solutions dedicated to the meat industry, electrospinning deserves special attention. This technique allows for the production of ultra-thin fibers from natural biopolymers (e.g., corn zein or pullulan). Nanofibers, which are loaded with essential oils and used as active spacers or inserts at the bottom of packaging trays. The extremely high surface area to volume ratio of nanofibers guarantees an extremely efficient and stable release of volatile substances into the free gas space inside the packaging, inhibiting the growth of molds and aerobic bacteria without direct contact of the liquid with the meat [80]. Additionally, co -encapsulation, which involves the simultaneous encapsulation of two different substances (e.g., rosemary oil and ascorbic acid) in a single nanocarrier, is becoming increasingly popular. This approach allows for a powerful synergistic effect, enabling a further reduction in the preservative dose and complete neutralization of undesirable sensory deviations in delicate meat products [81]. Essential oils were typically used at 0.1–1.0%, providing strong antimicrobial and antioxidant effects but risking sensory alterations [76]. Nanoemulsions (50–200 nm droplets) enabled 60% dose reduction while maintaining efficacy, lowering MDA by 20–40% and improving oxidative stability over 7–14 days of storage [81].

The study by Ghasemi et al. (2024) [82] aimed to develop an active edible coating designed to extend the shelf life of beef. The coating was formulated using carboxymethyl cellulose (CMC), okra mucilage (OM), zinc oxide (ZnO) nanoparticles, and nanostructured lipid carriers (NLCs) containing pennyroyal essential oil. Beef samples were treated with the coating and stored for 12 days at 4 °C. The researchers evaluated the material’s antimicrobial properties, microbial counts, pH, lipid oxidation (TBA) index, total volatile nitrogen (TVN) content, color parameters (L*, a*, b*), and the sensory characteristics of the meat. The results demonstrated a very high encapsulation efficiency for the essential oil (>98%) and significant antimicrobial activity of the coating. Samples treated with the nanocomposite material exhibited slower growth of total bacterial counts, lower pH, TBA, and TVN values, and better retention of the meat’s red color compared to the control group. Furthermore, the meat maintained higher sensory scores throughout the entire storage period. The authors concluded that the applied coating effectively inhibits microbial and oxidative spoilage processes in meat, thereby extending its refrigerated shelf life.

The study by Khalid et al. (2024) [83] focused on the development of a biodegradable packaging material based on carboxymethyl cellulose (CMC) enriched with nanoparticles containing pomegranate extract. Pomegranate polyphenols were nanoencapsulated within an alginate-chitosan system and subsequently incorporated into a carboxymethyl cellulose (CMC) matrix. The study evaluated the structural and mechanical properties of the resulting film, its antimicrobial activity against pathogenic bacteria, the release of active compounds, and the impact of the packaging on the quality of beef and poultry stored at 4 °C for 12 days. The results indicated that the synthesized nanoparticles had an average size of approximately 232 nm and a high encapsulation efficiency (87.98%). The film demonstrated strong antimicrobial efficacy against *Salmonella Typhimurium*, *Campylobacter jejuni*, *Staphylococcus aureus*, and *Listeria monocytogenes*. The use of this packaging material inhibited microbial growth, slowed down pH changes and lipid oxidation, and improved the sensory characteristics of the meat. Challenge tests showed a reduction in the counts of selected pathogens by as much as 2.6–5.0 log CFU/g. The authors concluded that CMC-based nanocomposite films containing pomegranate extract could serve as an effective, natural, and biodegradable active packaging solution for meat.

The study by Kutlu et al. (2024) [84] aimed to develop zein-based nanofibers containing flaxseed oil and gallic acid, and to assess their suitability for protecting poultry burgers during refrigerated storage. The nanofibers were produced via electrospinning and subsequently used as a coating material for meat products stored at 4 °C for 10 days. The antioxidant and antimicrobial properties of the nanofibers were evaluated, along with the encapsulation efficiency of the active ingredients, changes in the counts of total mesophilic aerobic bacteria (TMAB) and yeasts and molds (TYM), and the degree of lipid oxidation (TBA). The results confirmed a very high encapsulation efficiency for both flaxseed oil (97.1–98.0%) and gallic acid (91.5–92.5%). The coatings exhibited antioxidant and antimicrobial activity against selected pathogenic bacteria. The best protective effects were observed with nanofibers containing flaxseed oil (ZNF), which effectively inhibited the growth of bacteria and fungi and delayed oxidative processes. While control samples reached the limit of microbiological acceptability after just 3 days of storage, samples treated with the nanofibers maintained acceptable quality for up to 10 days. The authors demonstrated that zein-based nanofibers enriched with natural bioactive compounds can serve as an effective method for extending the shelf life of meat products by suppressing microbiological and oxidative processes.

The study by Wang et al. (2025) [85] aimed to develop a biodegradable, active absorbent pad for packaging chilled beef, composed of an aerogel based on cellulose nanocrystals derived from corn straw (CSCNCs) and acetylated starch (AS), enriched with a thymol nanoemulsion (TMO). The researchers analyzed the influence of varying ratios of CSCNCs to AS on the material’s structure, water absorption capacity, mechanical properties, thermal stability, thymol encapsulation efficiency, and the controlled release of thymol. Furthermore, the study evaluated the antimicrobial activity, biocompatibility, and biodegradability of the pads, as well as their effectiveness in extending the shelf life of chilled beef stored at 4 °C. The results indicated that an optimal structure was achieved with a CSCNCs:AS ratio of 1:5. An aerogel with this composition exhibited high porosity, significant mechanical strength, a water absorption capacity of approximately 2210%, and a high thymol encapsulation efficiency (90.35%). The release of the active compound was gradual and primarily governed by Fickian diffusion, which facilitated long-term antimicrobial action. Assessments of the material’s biological properties demonstrated effective inhibition of the growth of *Escherichia coli*, *Listeria monocytogenes*, and *Staphylococcus aureus*. The underlying mechanism involved the disruption of bacterial cell membranes by the released thymol. At the same time, the material demonstrated high biocompatibility with NIH 3T3 cells (a standard model line of mouse embryonic fibroblasts), confirming its potential safety for applications involving contact with food. The core of the study focused on evaluating the effectiveness of the pads during the storage of beef. Compared to commercial absorbent pads, the use of an aerogel containing thymol proved more effective in limiting the growth of the total viable count (TVC) of microorganisms, slowing the increase in total volatile basic nitrogen (TVB-N) levels and pH, and better preserving the color parameters of the meat. Beef protected by the new material maintained acceptable quality for approximately 12 days, whereas the shelf life of the control samples was about 8 days. The authors conclude that the developed aerogel is a promising active packaging material for chilled meat. The combination of high absorbency, controlled release of thymol, antimicrobial properties, and full biodegradability makes it possible to effectively extend the shelf life of beef while reducing the use of conventional plastics in food packaging.

## 5. Advanced Packaging Systems

Modern food packaging has evolved from passive barriers (protection from light and oxygen) to active and intelligent systems that extend shelf life and provide information on product quality. Table 3 summarizes advanced packaging systems for meat and meat products.

### 5.1. Active Packaging and Edible Coatings

Active packaging (AP) interacts with the contents or atmosphere within the package in a desired way. Two main approaches are used to preserve meat:

1. Scavenger systems—e.g., oxygen and moisture absorbers that inhibit the development of aerobes and reduce the rate of lipid oxidation.
2. Emitters systems—films and coatings that release antimicrobial and antioxidant substances directly onto the meat surface [95].

Active antioxidant packaging is a particularly dynamically developing sector, where traditional synthetic antioxidants (BHT, BHA) are replaced by polyphenols derived from byproducts of the agri-food industry. Incorporating extracts from green tea, grape skins, or fruit seeds into the polymer matrix of the film allows for the chemical binding of free radicals formed on the meat surface during its exposure to light in refrigerated display cases. Furthermore, the migration of these bioactive compounds to the surface layer of the raw material inhibits myoglobin oxidation, which directly translates into maintaining the desired, bright red color of fresh meat for a much longer period and reduces losses resulting from visual rejection by consumers [77]. There is a significant increase in interest in biofilms and edible coatings made from biopolymers (chitosan, gelatin, alginates, and polylactic acid—PLA) incorporated with natural bioactive agents or nanomaterials (e.g., zinc oxide ZnO, silver nanoparticles AgNP) [86,87,88,96]. The key technological challenge in designing such coatings is ensuring appropriate release kinetics of active substances to prevent their rapid depletion during the first days of storage. For this purpose, advanced biopolymer cross-linking methods are used, using non-toxic cross-linking agents such as citric acid or sodium tripolyphosphate. These agents create a dense, three-dimensional polymer network that slows the diffusion of essential oils and plant extracts, guaranteeing their uniform release at the minimum inhibitory concentration (MIC) level throughout the shelf life of the meat product [88]. To impart stronger biocidal properties to polymer coatings and matrices and improve their mechanical parameters, they are incorporated with nanomaterials, including zinc oxide (ZnO) nanoparticles, silver (AgNP), or nanoclays, creating so-called bionanocomposites. The addition of nanoparticles significantly reduces the film’s permeability to water vapor and oxygen, creating a barrier with thermomechanical and barrier properties similar to conventional polyolefin plastics, which facilitates the commercial implementation of these materials on packaging lines [89]. Excellent application results are achieved using chitosan, which, in addition to its role as a carrier, possesses intrinsic bactericidal properties resulting from its polycationic structure that damages bacterial cell membranes. For example, the application of a chitosan coating enriched with tea tree oil nanoemulsion on the surface of fresh chicken breasts allowed for extending their microbiological stability in refrigeration conditions from only 6 days to a record 16 days [87]. Biopolymer films (e.g., chitosan, alginate) containing 1–5% essential oils or plant extracts exhibited significant antimicrobial activity. Film thickness ranged from 50–150 μm. Application extended shelf life (e.g., from 6 to 16 days) and reduced TVB-N by 20–50% and MDA by 30–60%. Nanoparticles such as ZnO, AgNPs, and nanoclays were incorporated at 0.5–3%, improving oxygen barrier properties by 40–70% and mechanical strength by 20–50%. These films inhibited microbial growth and delayed oxidative changes [87,96].

The study by Chotprom et al. (2026) [97] was conducted to develop a biodegradable, intelligent packaging system for poultry meat that combines active product protection with the ability to monitor freshness. The packaging material was based on fish gelatin and sodium alginate, enriched with lemongrass essential oil and an anthocyanin-based indicator derived from *Clitoria ternatea* (butterfly pea) flowers. The system was tested on chicken breast fillets stored under refrigeration for 7 days. Evaluations included the physical and mechanical properties of the film, antimicrobial activity, microbiological parameters, pH, total volatile basic nitrogen (TVB-N) content, lipid oxidation (TBARS), drip loss, and changes in meat color. The results demonstrated that the addition of the essential oil effectively enhanced the protective properties of the packaging by inhibiting microbial growth and slowing down oxidative processes and protein degradation. Meat packaged in this active film exhibited lower TVB-N and TBARS values, reduced drip loss, and better retention of the desired color compared to the control group. Furthermore, the incorporated anthocyanin indicator allowed for the visual monitoring of changes during storage, as it changed color in response to rising pH levels and the accumulation of metabolites indicative of product quality deterioration. The authors conclude that the developed system can effectively extend the refrigerated shelf life of poultry meat while simultaneously providing an ongoing assessment of its freshness.

The study by Luo et al. (2026) [98] aimed to develop an active and intelligent packaging system for beef, combining antimicrobial functionality with the ability to monitor product freshness in real time. The system consisted of an active PCQ film—based on poly(vinyl alcohol) and chitosan (PVA/CS) and incorporating an oxidized tannin-copper complex (CuTAQ) with antibacterial properties—and an intelligent PCP indicator containing anthocyanins derived from purple sweet potatoes, which respond to pH changes and the presence of volatile amines generated during meat spoilage. The system’s efficacy was evaluated during the storage of beef at both refrigerated (4 °C) and room (25 °C) temperatures. The research analyzed the film’s mechanical, barrier, and hydrophobic properties, its UV-shielding capacity, its antimicrobial activity against *Escherichia coli* and *Staphylococcus aureus*, and the effectiveness of the colorimetric indicator in monitoring freshness. Additionally, the impact of the developed packaging on the shelf life and sensory quality of the stored beef was assessed. The results demonstrated that the inclusion of chitosan and CuTAQ significantly improved the film’s functional characteristics, enhancing its mechanical strength, barrier properties, and antibacterial activity. After 60 min of contact with the packaging material, *E. coli* and *S. aureus* cells were almost completely eliminated, confirming a broad spectrum of antimicrobial action. The use of an active film effectively slowed the spoilage process of beef, extending its shelf life to 48 h at 25 °C and up to 8 days at 4 °C, while maintaining desirable sensory characteristics. The intelligent PCP indicator demonstrated high sensitivity to pH changes and the presence of ammonia, allowing for a visual assessment of meat freshness through a distinct color change during storage. The observed color shifts correlated with the progressive degradation of the product, suggesting the indicator’s practical potential for real-time meat quality monitoring. The authors conclude that combining an antimicrobial active film with an intelligent freshness indicator represents an effective strategy for packaging beef. This system not only inhibits microbial growth and extends product shelf life but also enables consumers to perform a rapid, non-destructive quality assessment—thereby enhancing food safety and contributing to the advancement of modern intelligent packaging technologies.

The study by Ahmad et al. (2026) [99] aimed to develop a multifunctional, active, and intelligent packaging material designed to extend the shelf life of beef and monitor its freshness. The authors utilized a bilayer biopolymer matrix composed of gelatin, chitosan, and sodium alginate, enriched with MIL-100(Fe) metal–organic framework nanoparticles—which served as an antimicrobial agent—and beetroot extract containing betalains, which provided pH-responsive indicator properties. The research encompassed both the physicochemical characterization of the resulting films and an evaluation of their efficacy during the storage of beef. The analysis of the materials included an assessment of the structure and morphology of the nanofiller and the films, as well as their mechanical properties, barrier performance against UV radiation, oxygen, and water vapor, antioxidant activity, antimicrobial properties, and responsiveness to pH changes. Furthermore, the study explored the potential of using the film as a biosensor for meat freshness by observing color changes in the betalains, which correlate with the progressive spoilage of the product. The results demonstrated that the incorporation of MIL-100(Fe) into the biopolymer matrix improved the mechanical and barrier properties of the film while simultaneously imparting significant antimicrobial activity. Meanwhile, the presence of betalains enabled effective monitoring of beef quality through color changes triggered by an increase in pH and the accumulation of metabolites generated during meat spoilage. The films also exhibited significant antioxidant activity, which limited the rate of adverse oxidative changes occurring during product storage. Storage studies confirmed that the use of an active film containing MIL-100(Fe) and beetroot extract effectively slowed the deterioration of beef quality and extended its shelf life by approximately 3 days compared to a control biopolymer film lacking functional additives. At the same time, the system allowed for the ongoing assessment of the product’s freshness without the need for laboratory analysis. The authors conclude that combining MIL-100(Fe) nanostructures—which possess antimicrobial properties—with the natural pigment betalain enables the development of a new generation of intelligent, active packaging. Such a solution not only improves the microbiological safety and oxidative stability of meat but also allows for real-time monitoring of its freshness, representing a promising direction for the development of sustainable food packaging technologies.

### 5.2. Smart Freshness Indicators and Colorimetric Systems

Intelligent packaging (IP) is designed to monitor the condition of packaged food or its surroundings and communicate this information to the user [100]. Freshness indicators are crucial in the meat industry. During meat spoilage, bacterial enzyme activity and autolysis lead to protein degradation, the formation of volatile nitrogen bases (TVB N—Total Volatile Basic Nitrogen: ammonia, dimethylamine, trimethylamine) and a change in pH. [15,91,101]. Intelligent freshness indicators are most often colorimetric labels placed inside the packaging, containing halochromic dyes (sensitive to pH and the presence of TVB-N) [102]. The latest, pro-ecological trend is to abandon synthetic dyes in favor of natural plant extracts rich in anthocyanins (from purple sweet potatoes, red cabbage, and blackberries) or curcumin [103]. A change in the TVB-N concentration in the free space of the packaging causes an immediate, visible change in the color of the label (e.g., from red to pink to green-blue). This allows the consumer and distributor to absolutely, touchlessly verify the quality of the meat without having to open the packaging.

The basis of most modern colorimetric systems is the detection of volatile compounds released during food spoilage. In the case of meat, the key marker is the total content of volatile nitrogenous bases, including ammonia, dimethylamine, and trimethylamine. The release of these compounds causes a local pH change within the packaging, which is detected by the dye. Anthocyanins—natural pigments obtained from extracts of red cabbage, purple sweet potatoes, blueberries, and hibiscus flowers, among others—have gained particular popularity in the recent literature. As demonstrated by Yong et al. (2022) [90], anthocyanins incorporated into chitosan films exhibit an extremely sensitive, multicolored response to pH changes, ranging from red (acidic environment) through purple, to green and yellow (alkaline environment). Curcumin and betalains have also become alternatives to anthocyanins, which are characterized by different sensitivity profiles and higher thermal stability. Research conducted by Alizadeh -Sani et al. (2020) [91] shows that the use of mixtures of different natural dyes allows for precise calibration of the indicator for a specific type of food product. The key challenge in designing colorimetric indicators is to prevent the migration of the dye into the food and to ensure appropriate reaction kinetics. Traditional polymer films are currently being replaced by biopolymer matrices (starch, gelatin, agar, and pullulan). The use of nanotechnology, and in particular electrospinning, is a breakthrough in this field. Polymer nanofibrous mats are characterized by a huge surface-to-volume ratio, which significantly shortens the indicator’s response time. For example, electrospun Polyvinyl alcohol (PVA) nanofibers doped with plant extracts yield sensors capable of changing color in the presence of ammonia gas in just a few minutes [104]. Additionally, to improve the UV stability of the indicators, nanomaterials such as zinc oxide (ZnO) or cellulose nanocrystals (CNC) are incorporated, which protect the sensitive dyes from premature photochemical degradation [92]. In addition to TVB-N detection, carbon dioxide and hydrogen sulfide concentration indicators are being intensively developed, dedicated to products packaged in a modified atmosphere (MAP) and fresh poultry. In the case of detection CO_2_, the systems are based on reversible acid-base reactions in an aqueous medium immobilized in a hydrogel. Modern indicators H_2_S, emitted during the degradation of sulfur amino acids, often use biogenic silver nanoparticles (AgNPs) or copper, which, upon contact with hydrogen sulfide, undergo an irreversible reaction, changing color due to the formation of dark metal sulfides [105]. The latest innovation in colorimetric systems is eliminating the subjectivity of the human eye by integrating indicators with mobile applications. This phenomenon also fits into the concept of the Internet of Things. The colorimetric color code on the packaging is scanned using a smartphone camera, and a dedicated algorithm analyzes changes in the color space (e.g., RGB—Red, Green, Blue, CIELAB—Commission Internationale de l’Éclairage L\a\b\, or HSV—Hue, Saturation, Value). The application processes this data into specific information about shelf life (e.g., “fresh,” “use soon,” “spoiled”) [94]. The use of machine learning in the analysis of color indicators allows for highly accurate prediction of product spoilage kinetics in real time, which is invaluable for both consumers and cold chain managers. Halochromic indicators based on anthocyanins or curcumin embedded in PVA matrices responded to pH changes within 2–10 min, with visible color differences (ΔE > 5). Indicator response correlated strongly with TVB-N levels (R^2^ > 0.90), enabling real-time freshness monitoring [90,94].

The study by Xu et al. (2026) [106] aimed to develop a rapid, non-invasive system for assessing the freshness of raw meat, utilizing a colorimetric-fluorescent sensor array (CFA) combined with deep learning models. The sensor was constructed on a PVDF (Polyvinylidene Fluoride) membrane containing alizarin and FITC (Fluorescein Isothiocyanate), which react to the presence of volatile amines (NH_3_, trimethylamine, and dimethylamine)—indicators of meat spoilage. The system was tested on pork stored under refrigerated conditions, and the resulting images were analyzed using ResNet-152 and CMT-B neural networks. The research evaluated the sensor’s ability to detect biogenic amines, monitored changes in meat quality parameters—such as TVB-N, pH, TBARS, and total viable count (TVC)—and assessed the effectiveness of artificial intelligence models in classifying product freshness. A dataset of 8170 images, captured under both natural and UV light, was compiled, with samples classified as fresh, less fresh, or spoiled based on their TVB-N values. The results demonstrated high sensitivity of the CFA to fluctuating amine concentrations, with the response intensity increasing alongside exposure time and the levels of volatile compounds. The ResNet-152 model yielded the highest classification accuracy, reaching 95.82% under natural light and 97.88% under UV light. Additionally, a mobile application was developed that enables the assessment of meat freshness in approximately 130 ms, without the need for a connection to an external server. The authors concluded that combining smart chemical sensors with deep learning methods offers a promising tool for real-time meat quality monitoring and could be applied in modern intelligent food packaging systems.

Research by Wang and Takai (2026) [107] focused on the development of a smart gas sensor for monitoring the freshness of beef, based on a PVA/chitosan hydrogel containing a covalently bonded pH indicator—phenol red. The system was designed to detect volatile nitrogenous bases—primarily ammonia—produced during meat decomposition. Beef samples were stored under refrigeration for 14 days, and the sensor’s response was analyzed using machine learning techniques, including XGBoost and MLP models. The study evaluated the physicochemical properties of the hydrogel, its stability, selectivity toward various gases, operational repeatability, and the correlation between color changes and key indicators of meat freshness: total viable count (TVC) and total volatile basic nitrogen (TVB-N). Color analysis was performed within the CIE Lab* color space, and the resulting data were used to build machine learning-based predictive models. The results demonstrated that the developed hydrogel exhibited high chemical stability, no dye leaching, and strong selectivity for ammonia and other volatile amines. The sensor enabled the detection of very low ammonia concentrations (LOD = 0.0021%) and demonstrated high measurement repeatability. A strong correlation was found between the color change in the sensor and the TVC and TVB-N values. The best-performing regression model, based on the XGBoost algorithm, achieved a coefficient of determination (R^2^) of 0.94 for TVC and 0.99 for TVB-N, while classification models allowed for the differentiation between fresh, moderately fresh, and spoiled beef with an accuracy of up to 90%. The authors conclude that the developed system serves as an effective tool for the non-invasive, continuous monitoring of meat quality. Combining a stable hydrogel-based sensor with machine learning algorithms enables the precise assessment of both microbiological and chemical indicators of spoilage, offering promising prospects for the implementation of this technology in next-generation smart packaging.

## 6. Industry 4.0: Artificial Intelligence, Machine Learning and IoT

The modern meat industry is entering the era of digitalization, using Industry 4.0 tools to move quality control from the post-production level to a real-time predictive level [108].

### 6.1. Internet of Things (IoT) and the Digital Cold Chain

Maintaining an uninterrupted cold chain guarantees meat safety. The Internet of Things architecture enables continuous monitoring of environmental parameters (temperature, relative humidity, gas concentration) from the slaughterhouse, through logistics transport, to the store counters [109]. IoT sensors paired with wireless RFID tags (Radio Frequency Identification) and NFC (Near Field Communication) record the temperature history of each batch of goods. This data is immediately sent to the cloud. If a critical temperature is detected, the system generates an automatic alert. The use of blockchain technology additionally guarantees full transparency and traceability of the recorded data, which eliminates the possibility of falsifying the logistics history [110].

Traditional supervision of the cold chain of raw and processed meat products relies on passive temperature recording using autonomous devices (e.g., USB loggers), from which data is read post-factum, usually at the time of receipt of goods at the distribution center. This model makes it impossible to prevent damage to cargo. A technological breakthrough within the Industry 4.0 concept is the implementation of a digital cold chain (DCC) based on the Internet of Things architecture. Continuous, dynamic monitoring of environmental parameters in real time is crucial for meat processing, where even a short interruption of the cold chain leads to a rapid acceleration of the growth of spoilage microflora (including *Pseudomonas* spp., *Brochothrix thermosphacta*) and pathogenic (*Listeria monocytogenes*, *Salmonella* spp.), generating huge economic losses and the risk of epizootics [110]. The modern digital cold chain uses wireless sensor networks (WSN), active and semi-active RFID tags and BLE (Bluetooth Low Energy) beacons directly integrated with pallets, transport containers and elements of refrigerated wagons or trailers [111]. Modern IoT sensor nodes go beyond simple temperature measurement—they simultaneously record relative air humidity (RH), shocks and vibrations during transport, light level (detection of leaks in collective packaging) and gas concentration (CO_2_, O_2_, and ammonia) in the cargo space [111]. A significant technological challenge in meat logistics is the attenuation of radio signals (attenuation) caused by the high content of water and fat in the muscle tissue. This problem is solved by using low-power, long-range transmission protocols (LPWAN—Low-Power Wide-Area Network, e.g., LoRaWAN—Long Range Wide Area Network, and NB- IoT—Narrowband Internet of Things) combined with an edge computing architecture. Edge gateways placed in vehicles pre-filter and analyze local data streams, sending only key aggregates or alerts on crossing critical checkpoints (CCPs) to the cloud, thus reducing power consumption and bandwidth consumption [111].

The most advanced level of digitalization in cold chain logistics is the concept of a Digital Twin (DT) for a physical meat load. A Digital Twin is a virtual product model that processes continuous streams of telemetry data from IoT sensors in real time and subjects them to predictive microbiological models (e.g., Baranyi-Roberts models or Arrhenius equation) and fluid dynamics (CFD) simulations [112]. Instead of using a static expiration date (“best before”) printed at the slaughterhouse, a system powered by a Digital Twin calculates the dynamic remaining shelf life (RSL) in real time. If a micro-malfunction of the refrigeration unit and a local temperature increase are detected, the system automatically corrects the RSL of a given batch of meat. This allows for full resource allocation based on the FEFO (First-Expired, First-Out) strategy instead of the traditional FIFO (First-In, First-Out)—loads with a reduced RSL are automatically redirected by logistics algorithms to the nearest points of sale, eliminating food waste [110,112].

IoT sensor data provides a reliable legal and commercial basis for all supply chain participants (farmer, slaughterhouse, processor, carrier, and retail chain), it is integrated with distributed Blockchain ledgers [113,114] Telemetry data from IoT devices is automatically and irreversibly recorded in the blockchain, creating an unforgeable digital passport of the quality of a batch of meat [113]. The implementation of smart contract (SC) technology allows for the automation of receiving and financial procedures [114]. When the meat is delivered to the distribution center, the Blockchain system automatically verifies the full temperature history recorded by IoT sensors. If the permitted thermal threshold is exceeded during transport, the smart contract can immediately implement the procedure of automatic rejection of the goods, charge a contractual penalty to the carrier, or adjust the purchase price of the raw material without the need for lengthy court proceedings and laboratory expertise [113,114]. Sensor networks using RFID, NFC, LoRaWAN (868 MHz), or NB-IoT (LTE Band 20) recorded temperature and humidity every 1–5 min with accuracies of ±0.2 °C. Implementation reduced cold-chain losses by 15–30% and enabled dynamic shelf-life prediction (RSL) instead of static “best before” labeling [111].

### 6.2. Artificial Intelligence (AI), Machine Learning (ML), and Hyperspectral Imaging (HSI)

The integration of advanced analytical techniques with artificial intelligence is revolutionizing the assessment of meat freshness:

1. Hyperspectral Imaging (HSI)—combines the advantages of digital photography with spectroscopy, providing spatial and spectral information for each image pixel. HSI data is analyzed by machine learning algorithms (e.g., Convolutional Neural Networks—CNN, Partial Least Squares Regression (PLSR) allows for completely non-contact, non-destructive determination of water content, fat level, TVB-N index, myoglobin oxidation degree and detection of microbiological contamination in a few seconds [115,116].
2. Electronic nose (e-nose) and electronic tongue (e-tongue)—arrays of gas and electrochemical sensors imitate human sense organs. Data from the e-nose are analyzed by ML algorithms (e.g., Support Vector Machines—SVM, Random Forest) can distinguish the degree of meat freshness with an accuracy of >95% based on the profile of released volatile organic compounds (VOCs) [117,118].
3. Predictive AI Microbiology—Instead of relying on traditional, static expiration dates (“use by”), machine learning algorithms combine data from tags IoT (active temperature history) with kinetic models of microbiological growth. The result is a dynamic shelf life indicator that adjusts the expiration date depending on the actual storage conditions of the product [119].

HSI systems operated in the 400–1000 nm range with 5–10 nm spectral resolution. Machine learning models (CNN, PLSR) predicted TVB-N and microbial spoilage with 90–95% accuracy. VOC-based e-nose systems provided rapid contamination detection within 1–3 s, supporting real-time quality assessment [115,116,117].

## 7. The Influence of Meat Matrix Variables on the Effectiveness of Preservation Technology

The effectiveness of modern meat preservation methods is strongly influenced by the properties of the meat matrix itself. In particular, the type of meat, fat content, and initial microbiological load determine both the physical and biochemical processes and the final technological outcome. The results of the literature analysis indicate that these three variables significantly interact with the mechanisms of action of all the technologies described in this review. Table 4 presents the influence of variables related to the meat matrix on the effectiveness of selected preservation technologies.

Meat type as a factor determining the response to preservation technology

The type of meat influences protein structure, water content, enzyme activity, and susceptibility to oxidation, which translates into a varied response to non-thermal technologies. In the case of HPP, red meats exhibit a marked susceptibility to myoglobin denaturation, as confirmed by the observation that pressures above 300 MPa induce myoglobin denaturation and heme group release. Poultry, on the other hand, due to their higher PUFA content, is more susceptible to lipid oxidation when exposed to RONS generated in ACP [32,33]. In mechanical technologies (HIU, PEF), the structure of muscle fibers plays a key role. Poultry, with its more delicate structure, undergoes texture changes more quickly under the influence of cavitation, while beef requires higher pulse intensity to achieve fiber crushing [127]. In the case of PL, effectiveness depends on surface topography—uneven poultry elements increase the “shadowing” effect, limiting the penetration of light pulses [51].

Fat content and susceptibility to oxidation and energy absorption

High fat content physically protects bacterial cells from mechanical damage during HPP treatments and isolates them from the current flow in PEF technology, which necessitates the use of higher process parameters. In technologies based on strong oxidative stress or free radical generation (ACP, HIU, and PL), fat degrades rapidly. The fattier the meat, the narrower the technological window—the process must be shorter to avoid critical rancidity [127,128]. Fat reduces the effectiveness of natural essential oils and plant extracts. Hydrophobic active substances (e.g., carvacrol) migrate into the fat matrix, drastically reducing their concentration in the aqueous phase (where bacteria live and reproduce) [78,121]. Fat is one of the key factors modulating the effectiveness of preservation technologies, particularly those generating free radicals or light energy. In HPP, fatty meats exhibit greater susceptibility to lipid peroxidation, which is consistent with the observation that high pressures can induce lipid peroxidation involving free iron [32]. A similar effect is observed in HIU, where the generated OH^•^ radicals can initiate reactions leading to acceleration and intensification of radical rancidity [39,40]. In ACP, fat reacts intensively with RONS, which accelerates PUFA oxidation and requires shorter exposure time to limit sensory degradation. In optical technologies (PL), fat absorbs some light energy, reducing photodecontamination efficiency. In PEF, however, fat reduces electrical conductivity, reducing electroporation efficiency [127]. In magnetic field-assisted freezing (RMF/OMF), fat hinders the uniform formation of ice crystals, which can increase post-thaw leakage, particularly in pork and beef. For example, lean meat can be effectively maintained in a supercooled state using PEF alone, whereas fatty meat requires the simultaneous use of PEF + OMF to maintain a stable supercooled state [58].

Initial Microbial Load and Process Requirements

High initial microbial contamination significantly increases the demands on the preservation technology. Higher pressures are necessary in HPP to achieve >5-log_10_ reduction, as evidenced by data showing that 600 MPa allows for greater than 5-log_10_ cfu/g reduction in *L. monocytogenes* [32,33]. In HIU and ACP, a high load requires longer exposure, which increases the risk of undesirable oxidative changes [39,46]. In Poland, high levels of surface contamination promote photodecontamination efficiency, but the shadowing effect remains a significant limitation. In PEF, a higher number of pulses can affect meat texture, especially in low-conductivity products [51,52,53]. In biopreservation, high microbial loads require synergistic strategies, based on the “hurdle technology” principle. Bacteriocins have limited efficacy against Gram-negative bacteria, so they are combined with HPP or essential oils, which destabilize the pathogens’ cell membranes, sensitizing them to antimicrobial peptides. Bacteriophages and endolysins remain effective even with high contamination levels, but require carefully selected phage cocktails [70,71].

Interactions between matrix variables and fixation technologies

The analysis indicates that meat matrix variables do not act independently but interact in complex ways with the technology mechanisms. Fatty and red meats are more susceptible to oxidation and color changes, poultry is sensitive to RONS and PL, and high microbial loads require more intensive process parameters. In industrial practice, this means that the technology must be individually optimized for each meat matrix, according to the principle of “synergistic combination of mild fixative factors” [129,130,131,132,133,134]. Table 5 presents an overview of experimental studies on the application of “hurdle technology”in meat and meat products.

## 8. Implications for Industry and Consumers

The rapid development of the described innovations has wide practical consequences:

An industrial perspective

Integrating HPP, ACP, and IoT technologies reduces returns and financial losses resulting from spoilage. Digital supply chain optimization significantly reduces food waste, bringing the industry closer to sustainable development (ESG—Environmental, Social, and Governance) requirements. However, implementing these solutions involves high upfront costs (CAPEX—Capital Expenditure), especially in the case of high-pressure lines or HSI, and requires the employment of highly skilled engineering staff to operate ML/IoT systems.

Throughput Limitations and Batch Challenges

The main problem with implementing new-generation physical methods is the mismatch between the kinetics of the preservation process and the capacity of modern, continuous meat cutting, portioning, and packaging lines (reaching 100–200 packages per minute). HPP pressure reactors operate in a strictly cyclical (batch) mode. The full processing cycle (loading baskets, filling with isostatic water, increasing pressure to 300–600 MPa, holding time for 3–5 min, depressurization, and unloading) takes 6 to 10 min. This discrete nature of operation creates bottlenecks in the production line. To maintain packaging continuity, plants must use expensive refrigerated storage buffers or invest in sets of several HPP reactors operating in parallel, which multiplies capital expenditures [29,127,128,138].

Optical and surface constraints in pulsed light (PL)

PL technology achieves high levels of decontamination in fractions of a second, but its throughput is limited by the geometry of the meat surface. The “shadow” phenomenon on porous, irregular muscle tissue necessitates the use of complex systems of multi-directional lamps at the top and bottom of the conveyor belt. Increasing the belt speed shortens the exposure time, which necessitates an increase in the flash frequency (Hz) or dose energy, leading to excessive surface heating of the meat and photodegradation of lipids [138].

Cold plasma diffusion time (ACP) and in-pack pasteurization

When cold plasma is generated directly inside a sealed package, the generation of reactive oxygen and nitrogen species (RONS) occurs rapidly, but their effective diffusion into the microcracks of the meat requires a specific reaction time. Scaling this process requires extending the buffer conveyor lines before the refrigeration line to ensure adequate contact time of the biocidal gases with the matrix before their natural decomposition [139,140].

Barriers to scaling process parameters

Transferring process parameters from small laboratory samples (e.g., Petri dishes or 20 g meat slices) to 5–25 kg meat blocks or whole carcasses raises complex physicochemical issues.

Wave attenuation in high-intensity ultrasonication (HIU)

The destruction of cellular structures and enhanced curing using HIU is based on the phenomenon of cavitation. However, the power density of the ultraacoustic wave decreases exponentially with tissue penetration depth due to the high attenuation coefficient of muscle and fat. Differences in acoustic impedance between fat and connective tissue lead to uneven energy absorption—zones of overheating develop in the bottom regions (generating OH^•^ free radicals and rancidity), while deeper layers of meat remain unprocessed [141].

Uniformity of micro-discharges in cold plasma (ACP)

On an industrial scale, maintaining a stable dielectric barrier discharge with varying gas free volume in large MAP trays is difficult. Localized electric micro-arcs occur, which, instead of generating cold plasma, generate local high-temperature points, leading to burning of the packaging film or local denaturation of myogenic proteins [139].

Low electrospinning efficiency for nanofibers

Halochromic indicators or nanofibril cartridges releasing essential oils by axial electrospinning achieves throughputs of several milligrams to grams per hour at laboratory scale. Scaling up to mass scale requires multi-nozzle needleless devices, which pose challenges related to maintaining a constant electric field (e.g., 20–50 kV), susceptibility to fluctuations in relative humidity on the production floor, and the risk of solvent vapor explosion [93].

Barriers to regulatory approval and certification

The introduction of biopreservatives, bacteriophages, and nanomaterials into the food market (especially in the European Union) is subject to stringent legal regulations (EFSA/FDA—European Food Safety Authority/Food and Drug Administration). Although phase preparations (e.g., those directed against *L. monocytogenes*) have GRAS (Generally Recognized As Safe) status in the US, their widespread use in the European Union encounters legislative resistance. EFSA requires clear evidence that the lytic phages used do not possess toxin-encoding genes and are not capable of horizontally transferring antibiotic resistance genes to the consumer’s gut microflora. The use of isolated endolysins or bacteriocins in their pure form requires registration as food additives (with an E number) or classification as a Novel Food, which requires several years of multi-million dollar toxicological studies [142].

Migration of nanomaterials from packaging (ZnO, AgNPs, and Bionanocomposites) zinc oxide (ZnO) or silver nanoparticles (AgNPs) into packaging films to improve barrier and biocidal properties is regulated by Regulation (EC) No. 1935/2004 [143] and Regulation (EU) No. 10/2011 [144] on plastic materials intended to come into contact with food. The main barrier is specific migration limits (SMLs). Particles < 100 nm in size can diffuse into acidic or fatty meat simulants. Due to the risk of accumulation in internal organs and generation of oxidative stress in the intestinal epithelium, EFSA applies very strict acceptance criteria [145].

Clean Paradox Label

The use of purified plant extracts or isolated bacteriocins for preservative purposes may, in accordance with legal regulations, require marking them on the label as a “preservative”, which eliminates the marketing goal of implementing clean technology label. To avoid this, the industry tends to use them under the names “aromatic extracts” or “starter cultures,” which in turn is sometimes questioned by trade inspectors if a deliberate biocidal effect is demonstrated [142].

Compatibility with existing meat processing lines

Implementing new preservation systems in production plants that have been operating on the market for a very long time requires their integration without revolutionizing the process chain. Industrial HPP devices weigh from several to several dozen tons and require reinforced foundations, dedicated high-pressure hydraulic systems, and extensive glycol cooling systems. Many existing processing plants lack adequate ceiling load-bearing capacity or free space in clean areas. Fresh meat processing must be performed at a temperature of 0–4 °C. Meanwhile, pulsed light (PL) systems generate large amounts of waste heat from xenon lamps, and HPP pasteurization causes a temperature increase due to adiabatic compressibility (approximately 3 °C per 100 MPa). If meat exposed to 600 MPa heats up from 4 °C, 22 °C, the immediate implementation of efficient cooling tunnels before packaging becomes necessary, which increases electricity consumption and negatively impacts the technology’s environmental impact [138].

Integration of active and intelligent films with packaging machines

Advanced bionanocomposites (e.g., polylactic acid (PLA), chitosan, or starch) have different elasticity, tensile strength, and sealing temperature compared to standard PE/PP/PA (polyethylene/polypropylene/polyamide) laminates. Using them on high-speed thermoforming machines or flow-pack packaging machines can lead to film jams, seam breakage, and loss of MAP seal integrity. Halochromic labels (freshness indicators) are susceptible to mechanical damage during automatic labeling and the risk of false readings caused by condensation inside the packaging [146].

Odor cross-contamination and equipment cleaning (CIP—Cleaning -in-Place). The use of essential oils (oregano, thyme) as sprays or release coatings results in the deposition of volatile aromatic compounds (e.g., carvacrol, thymol) on conveyor belt components and nozzles. This requires modifications to CIP cleaning and disinfection procedures to prevent the transfer of intense herbal odors to subsequent, delicate product batches (e.g., fresh poultry) [60].

Consumer perspective

The introduction of “clean labels,” biopreservation, and halochromic indicators directly impacts consumer health and safety. The elimination of carcinogenic nitrates and the assurance of freshness, visually confirmed by smart labels, increase product confidence. However, the “price premium” poses a challenge—innovatively packaged premium products tend to be more expensive, which can be a barrier to demand in times of inflation.

Food safety and nanomaterial migration risks—knowledge gaps

Despite the promising technological properties of nanoencapsulated plant extracts and bionanocomposite packaging materials—such as effective protection of bioactive compounds, masking intense aromas, and controlled release of active substances [63,79]—their use in meat matrices still raises significant concerns about consumer safety. Current knowledge is clearly incomplete, as reliable toxicological data are lacking, and most studies focus on antimicrobial effectiveness, omitting key issues related to nanoparticle migration into food and their behavior in the gastrointestinal tract. The industrial use of bionanocomposites (e.g., those containing silver nanoparticles AgNPs, zinc oxide ZnO, or nanoclays) and lipid nanocarriers raises legitimate questions about consumer health safety. The literature still lacks comprehensive long-term (in vivo) studies analyzing the fate of released nanostructures and ions after their penetration from the packaging into the meat matrix and subsequent human consumption.

The main knowledge gaps include [144,147,148,149]:

1. Migration models are inaccurate. Standard tests are performed in liquid simulants (e.g., 3% acetic acid or 10% ethanol). Accurate models describing the kinetics of ion diffusion Ag^+^ in Zn^2+^ the complex, solid protein-fat matrix of fresh meat during storage in the cold chain are lacking.
2. Accumulation and oxidative stress. Although zinc is an essential trace element for the body, excessive local release of ions Zn^2+^ or ZnO nanoparticles and AgNPs < 100 nm can generate reactive oxygen species (ROS), leading to oxidative stress and damage to cell membranes in the gastrointestinal epithelium.
3. Modification of the intestinal microbiota. Due to the strong antibacterial properties of nanoparticles, their uncontrolled entry into the gastrointestinal tract may inhibit the growth of beneficial intestinal microorganisms (e.g., *Lactobacillus* or *Bacteroides*), causing dysbiosis.
4. Analytical challenges and regulatory constraints. Routine quality control is hampered by the lack of cost-effective and rapid analytical methods to distinguish free nanoparticles in meat tissue from ions released from packaging materials, which complicates verification of compliance with specific migration limits (SMLs).

## 9. Limitations of This Review

Despite the thorough methodological approach, the presented review paper has certain limitations:

1. Database and language barrier. The study relied on selected commercial resources (Scopus, PubMed, WoS, ScienceDirect) and English-language publications. This may result in missing innovative research results from Asia (published in Chinese) or Spanish-speaking countries, which are important meat producers.
2. Industrial secrecy is a matter. Many cutting-edge AI algorithms for optimizing cold chains and active ingredients in packaging films are heavily protected by corporate patents. They are not widely available in peer-reviewed, open-access journals.
3. Dynamic progress in IT. The development of artificial intelligence, including language models, is so rapid that some of the information published between 2021 and 2023 regarding advanced e-noses may have become outdated during implementation in favor of even newer Edge AI solutions.

The restriction of the search to English-language publications and major commercial databases (Scopus, Web of Science, PubMed, ScienceDirect) introduces a potential selection bias that may subtly shape the synthesized conclusions of this review. Specifically, omitting regional literature—particularly from dominant meat-producing and exporting regions such as East Asia and Latin America—may lead to an underestimation of localized biopreservation practices, regional regulatory frameworks, and pragmatic, low-cost technological adaptations. Furthermore, indexed literature predominantly reflects high-resource academic environments, which could convey an overly optimistic perception of the commercial readiness level and economic feasibility of capital-intensive technologies (e.g., AI-driven Digital Twins, hyperspectral imaging, and electrospun nanofibrous indicators). Consequently, while the fundamental mechanisms and overarching technological trends identified in this study remain scientifically sound, the generalized conclusions regarding global industrial adoption rates and market integration should be interpreted within the context of these selection boundaries.

## 10. Conclusions

The preservation of meat and meat products at the beginning of the second half of the 21st century is based on the paradigm of sustainable development, the abandonment of chemical preservatives, and the full digitalization of the supervision process. The success of the “hurdles” concept lies in the synergy of mild, non-thermal technologies (HPP, ultrasound, and cold plasma) and biopreservation (LAB cultures, nanoencapsulated phytoextracts, and bacteriophages). This approach is a response to the uncompromising requirements of the “clean label” trend, protecting the nutritional and sensory qualities of meat. Material innovations are complemented by active coatings and intelligent, colorimetric freshness indicators based on natural dyes. Furthermore, the implementation of IoT infrastructure, hyperspectral imaging, and machine learning is revolutionizing supply chains, transforming static quality inspection into predictive, multidimensional, real-time analyses. The full commercialization of these integrated systems is key to improving global food security and dramatically reducing food waste. However, the commercialization of innovative meat preservation methods requires moving away from treating them as standalone units in favor of a hybrid approach (“hurdle technology”), where reduced physical parameters (e.g., HPP at 200 MPa combined with natural antioxidants) are integrated with real-time digital parameter control (IoT/AI).

## Figures and Tables

**Table 1:** Comparison of selected leading non-thermal meat preservation technologies.

Technology	Main Effects and Advantages	Disadvantages and Limitations	Refs.
HPP (High Pressure Processing)	Inactivation of pathogens; shelf-life extension; absence of high temperatures.	High investment costs; risk of color change and lipid oxidation.	[12,13,14]
HIU (High-Intensity Ultrasound)	Reduction in curing time; surface decontamination; improvement of tenderness.	Potential generation of free radicals leading to lipid rancidity.	[15,16,17,18,19]
ACP (Atmospheric Cold Plasma)	High biocidal efficacy; clean technology with no chemical residues.	Limited penetration depth; impact on quality indicators (e.g., color) under prolonged exposure.	[20,21,22]
PL (Pulsed Light)	Instantaneous (second-level) microbial reduction.	“Shadowing” phenomenon on porous/uneven surfaces.	[23]
PEF (Pulsed Electric Fields)	Membrane electroporation; accelerated marinade diffusion; structural tenderization.	Requirement for a medium with appropriate conductivity.	[24]

**Table 2:** Overview of meat biopreservation techniques.

Technique/Active Compound	Achieved Technological and Safety Effects	Industrial Limitations and Challenges	Ref.
Bacteriocins (e.g., from LAB cultures)	Protection against foodborne pathogen growth in the matrix; preservation of the product’s natural character.	Sensitivity to matrix environmental conditions (pH, presence of proteases) and a narrow spectrum against Gram-negative bacteria.	[13]
Bacteriophages	Highly specific eradication of target pathogens; complete lack of impact on the aroma and taste of the meat matrix.	Requirement for precise design of specific cocktails due to the highly restricted host range of bacteria.	[12]
Essential oils in general (e.g., oregano, thyme)	Very strong biocidal properties disrupting cell membranes of pathogenic and spoilage bacteria.	High aroma intensity, which even at low concentrations can disrupt the desired meaty sensory profile of the product.	[60]
Active packaging with essential oils	Gradual, continuous release of active compound vapors onto the meat surface; strong mold inhibition.	Technological complications in film manufacturing; migration of intense aromas into the target product.	[61]
Plant extracts (e.g., green tea, rosemary, cinnamon)	Excellent antioxidant properties; delay of oxidation and color loss; clean label status.	Depending on dosage—risk of tissue darkening, astringent taste, or distinct sweet-spicy off-flavor.	[62]
Nanoencapsulation of extracts and essential oils	Masking of dominant herbal odors, protection of antioxidants, and their controlled release into food.	Complex manufacturing process of delivery systems (e.g., nanoliposomes) and consumer distrust toward the term “nanotechnology”.	[63]

**Table 3:** Overview of advanced packaging systems for meat and meat products.

Packaging Technology	Main Advantages	Main Disadvantages/Technological Challenges	Refs.
Active scavenger packaging (e.g., oxygen and moisture absorbers)	Limit aerobic bacterial growth and inhibit lipid autoxidation; improve shelf-life stability without directly interfering with the chemical composition of the meat.	Limited absorption capacity; lack of active release of biocidal substances to directly combat pathogenic microflora.	[86]
Active antioxidant packaging (emitters) with plant extracts	Replace synthetic antioxidants (clean label); effectively neutralize free radicals, inhibit myoglobin oxidation, and maintain the desirable red meat color.	Need for precise control of diffusion kinetics to prevent premature depletion of polyphenols; risk of altering the product’s flavor/aroma profile.	[77,86]
Edible coatings and biodegradable films (PLA, gelatin, alginates, chitosan)	Complete biodegradability; chitosan coatings exhibit intrinsic, strong antibacterial properties (up to 16 days of cold storage stability) and reduce drip loss.	Require advanced cross-linking (e.g., with citric acid) to prevent overly rapid film degradation and burst release of active substances during the initial days.	[86,87,88]
Bionanocomposites (films doped with nanomaterials: ZnO, AgNPs, and nanolays)	Provide a strong barrier to water vapor and oxygen; significantly improve the thermo-mechanical properties of biopolymers, approaching those of conventional polyolefins.	Need for strict monitoring of potential heavy metal nanoparticle migration into the meat matrix; high manufacturing costs.	[87,89]
Intelligent colorimetric indicators (with natural halochromic dyes)	Non-destructive, continuous freshness monitoring (TVB-N concentration and pH); natural dyes (anthocyanins, curcumin) are consumer-safe; clear visual signal (color change).	Susceptibility of sensitive dyes to photochemical degradation (UV) and oxidation; risk of dye migration into meat; subjectivity of visual shade evaluation by the customer.	[15,90,91]
Nanofibrous indicator matrices (Electrospinning)	Extremely high surface-area-to-volume ratio guarantees an ultrashort response time to gases (on the order of minutes); excellent dye immobilization in the PVA (polyvinyl alcohol) matrix.	Complex scaling process of electrospinning technology from laboratory level to mass industrial production; low mechanical strength of the nanofibers themselves.	[91,92,93]
Smartphone-integrated indicators (IoT and AI)	Complete elimination of visual assessment subjectivity; precise, quantitative calculation of volatile basic nitrogen concentration and accurate shelf-life forecasting.	Requires a smartphone with a dedicated app installed and appropriate lighting conditions to take a reliable photo of the color indicator.	[94]

**Table 4:** The influence of variables related to the meat matrix on the effectiveness of selected preservation technologies.

Technology	Influence of the Type of Meat	Effect of Fat Content	Influence of Initial Microbial Load	Refs.
HPP	Red meats: severe myoglobin denaturation; poultry: greater susceptibility to oxidation	Fat enhances pressure-induced lipid peroxidation	High load requires >500 MPa for >5 log reduction	[32,33]
HIU	Poultry: rapid texture changes; beef: requires more acoustic energy	High fat content accelerates and intensifies radical rancidity	High charge requires longer exposure, increasing the risk of oxidation	[39,40]
ACP	Poultry more susceptible to color changes; red meats sensitive to RONS oxidation	Fat reacts strongly with RONS, accelerating the oxidation of PUFAs	Gram-negative bacteria -are more difficult to inactivate at high contamination levels	[44,45,46,120]
PL	Uneven poultry surfaces increase the shadowing effect	Fat absorbs some of the energy of the PL, reducing its effectiveness	High charge increases PL efficiency but is limited by surface topography	[49,50,51]
PEF	Higher conductivity of poultry meat improves electroporation	Fat lowers conductivity, reducing efficiency	A high charge requires more pulses, which affects the texture	[52,53]
RMF/OMF	Fatty beef requires simultaneous application of PEF + OMF to maintain a stable supercooled state	Fat interferes with the even formation of ice crystals, increasing leakage.	High charge limits effectiveness; magnetic fields do not destroy biofilms	[58]
Biopreservation (LAB, bacteriocins)	pH and enzyme activity vary between species, affecting the stability of bacteriocins.	Fat binds active compounds, reducing their availability	High payload requires a hurdle strategy (e.g., HPP + LAB).	[67,68,69]
Bacteriophages/endolysins	Effectiveness depends on the dominant pathogen in a given meat	Fat has minimal effect	High load requires precisely selected phage cocktails	[70,71]
Plant extracts/essential oils	Poultry more susceptible to sensory changes in monoterpene aroma	Fat binds carvacrol and thymol, reducing antimicrobial activity	High load requires higher doses → risk of sensory defects	[78,121]
Nanoencapsulation	Facilitates application in meat with a delicate flavor profile (poultry)	Protects active compounds from oxidation in fatty meat	Effective even with high contamination thanks to controlled release	[82,83,84,85]
Active packaging	Poultry is very sensitive to aroma migration from active films; red meats are more tolerant	Fat absorbs volatile active substances (e.g., EO—essential oils, vapors), reducing the effectiveness	High charge requires stronger release kinetics of active compounds	[97,98,99]
Edible coatings	Coatings adhere differently depending on the surface structure (poultry vs. beef)	Fat hinders the adhesion of the coating and slows down the diffusion of active substances	High charge requires thicker coatings or nanoparticle reinforced matrices	[122,123]
Electrospinning	Nanofibers Electrospun can be used as coatings or contact liners; the sensory sensitivity of poultry requires controlled release of active substances	Fat influences the migration of volatile compounds from nanofibers; in fatty meat, modifications of the carrier matrix are necessary	High microbiological load requires combination with other methods (e.g., nanofibers -+ HPP) and control of the antiseptic release kinetics	[124,125,126]
Freshness indicators (halochromic, nanoparticles)	Poultry spoils faster → color change faster; red meats may mask the color signal	Fat slows down the formation of TVB-N → delayed indicator response; fat may affect color perception	High charge speeds up the indicator response; a rapid increase in TVB-N causes an intense color change	[106,107]

**Table 5:** Experimental studies using the “hurdle technology” in meat and meat products.

Meat/Product	Technologies/Factors	Technological Parameters	The Authors’ Assumptions	Authors’ Conclusions	Ref.
Raw beef	Cooling + MAP + antioxidants	MAP (Modified Atmosphere), Natural Antioxidants, 4 °C Cooling	To test whether combining several mild factors will improve microbiological safety, oxidative stability and beef quality more than single methods	The synergy of “hurdles” limits microbial growth, reduces lipid oxidation and improves sensory quality; an effect stronger than the action of individual factors	[130]
Pork salami (nitrite-free)	Fermentation + low temperature ripening + aw control	LAB (Lactic Acid Bacteria) at low temperature, RH (Relative Humidity) 65–80%, and w ≤ 0.90	To assess whether a set of mild agents can replace nitrites in salami production while maintaining safety and quality.	Possible production of nitrite-free salami while maintaining microbiological stability; key parameters are RH and aw	[131]
Fermented sausage (model)	HHP + bacteriophage P100 + bacteriocinogens *Pediococcus acidilactici*	HHP 300 MPa, bacteriophage Listex ™ P100, pediocin PA-1	Test whether the combination of HHP, bacteriophage and bacteriocins can effectively inactivate *Listeria monocytogenes*	Strong synergistic effect against *Listeria monocytogenes*; hurdle system (HHP + P100 + bacteriocins) more effective than single methods	[132]
Pepperoni (red meat)	Salt + nitrites + heat treatment + aw control	Salt/nitrite reduction, aw control, heat treatment parameters	pepperoni safety and quality and the potential for salt/nitrite reduction	Synergy of factors allows for salt and nitrite reduction while maintaining safety; effects dependent on process stage	[133]
Red meat (slices)	Active composite film + mild UV	Citrus peel inspired composite film + mild UV (short exposure)	Check if active film + UV can act as a hurdle system to extend the refrigeration shelf life of meat	The combination of active film and UV extends refrigeration shelf life, limits the growth of spoilage bacteria and does not deteriorate sensory quality	[129]
Chicken breast (poultry)	Solid essential oils (non-contact EO) + MAP	Solid EO (spice EO), MAP, cooling	To test whether solid EO + MAP will extend the refrigeration life of chicken and change the microflora without direct contact with the meat	EO + MAP extends refrigeration shelf life, limits the growth of spoilage bacteria and modifies microflora; the hurdle system enables a “clean-label”	[134]
Chicken breast (poultry)	Electrospun nanofibers + natural extract (TVB N index -)	nanofibers + plant extract	Develop an intelligent TVB-N indicator for chicken freshness	Effective detection of TVB-N and NH_3_; real-time monitoring	[135]
Red meat (beef)	PLA-nanofibers electrospun + anthocyanins (spoilage indicator)	PLA nanofibers + anthocyanins	Develop a colorimetric indicator for beef spoilage	Sensitive detection of NH_3_ and TVB-N; distinct color change	[136]
Red meat (beef)	Biodegradable Active Smart Packaging + Nanofibers electrospun	Starch + nanofibers electrospun	Develop active packaging with a freshness indicator	Extended shelf life, effective freshness monitoring	[137]

## Data Availability

No new data were created or analyzed in this study. Data sharing is not applicable to this article.

## Abbreviations

The following abbreviations are used in this manuscript:

ACP	Atmospheric Cold Plasma
AgNP/AgNPs	Silver Nanoparticles
AI	Artificial Intelligence
anti-QS	Anti-Quorum Sensing
AP	Active Packaging
APC	Aerobic Plate Count
BHA	Butylated Hydroxyanisole
BHT	Butylated Hydroxytoluene
BLE	Bluetooth Low Energy
CAP	Cold Atmospheric Plasma
CAPEX	Capital Expenditure
CCP	Critical Control Point/Critical Checkpoint
CFA	Colorimetric/Fluorescent Array
CFD	Computational Fluid Dynamics
CFS	Cell-Free Supernatants
CFU	Colony Forming Units
CNC	Cellulose Nanocrystals
CNN	Convolutional Neural Networks
CRISPR-Cas	Clustered Regularly Interspaced Short Palindromic Repeats and CRISPR-Associated Proteins
DBD	Dielectric Barrier Discharge
DCC	Digital Cold Chain
DT	Digital Twin
EO	Essential Oil
ESG	Environmental, Social, and Governance
FAO	Food and Agriculture Organization of the United Nations
FEFO	First-Expired, First-Out
FIFO	First-In, First-Out
FITC	Fluorescein Isothiocyanate
HIU	High-Intensity Ultrasound
HPP	High Hydrostatic Pressure/High Pressure Processing
HSI	Hyperspectral Imaging
IoT	Internet of Things
IP	Intelligent Packaging
LAB	Lactic Acid Bacteria
LPWAN	Low-Power Wide-Area Network
MAP	Modified Atmosphere Packaging
MDA	Malondialdehyde
MIC	Minimum Inhibitory Concentration
ML	Machine Learning
NFC	Near Field Communication
NH_3_	Ammonia
OMF	Oscillating Magnetic Field
PAA	Peracetic Acid
PEF	Pulsed Electric Fields
PFU	Plaque Forming Units
PL	Pulsed Light
PLA	Polylactic Acid
PLSR	Partial Least Squares Regression
PUFA	Polyunsaturated Fatty Acids
PVA	Polyvinyl Alcohol
PVDF	Polyvinylidene Fluoride
QS	Quorum Sensing
RFID	Radio Frequency Identification
RH	Relative Humidity
RMF	Rotating Magnetic Field
RONS	Reactive Oxygen and Nitrogen Species
RSL	Remaining Shelf Life
RTE	Ready-To-Eat
SC	Smart Contract
SVM	Support Vector Machines
TBARS	Thiobarbituric Acid Reactive Substances
TBHQ	Tert-Butylhydroquinone
TVB-N	Total Volatile Basic Nitrogen
TVC	Total Viable Count
TVN	Total Volatile Nitrogen
UV	Ultraviolet
UV-C	Ultraviolet C
VOCs	Volatile Organic Compounds
WBSF	Warner–Bratzler Shear Force
WSN	Wireless Sensor Networks
ZnO	Zinc Oxide
